# IRES inhibition induces terminal differentiation and synchronized death in triple-negative breast cancer and glioblastoma cells

**DOI:** 10.1007/s13277-016-5161-4

**Published:** 2016-07-26

**Authors:** Christos Vaklavas, William E. Grizzle, Hyoungsoo Choi, Zheng Meng, Kurt R. Zinn, Kedar Shrestha, Scott W. Blume

**Affiliations:** 1 Comprehensive Cancer Center, University of Alabama at Birmingham, Birmingham, AL 35294 USA; 2 Department of Medicine, Division of Hematology/Oncology, University of Alabama at Birmingham, Birmingham, AL 35294 USA; 3 Department of Pathology, University of Alabama at Birmingham, Birmingham, AL 35294 USA; 4 Department of Pediatrics, Seoul National University Bundang Hospital, Gyeonggi-do, 463-707 South Korea; 5 Department of Biochemistry and Molecular Genetics, University of Alabama at Birmingham, Bevill Biomedical Research Bldg Room 765, 845 19th Street S, Birmingham, AL 35294 USA; 6 Analytical Development Division, Novavax Inc., Rockville, MD 20850 USA; 7 Department of Radiology, University of Alabama at Birmingham, Birmingham, AL 35294 USA

**Keywords:** Triple-negative breast cancer, Glioblastoma, Terminal differentiation, Quorum-sensing, Hill slope coefficient

## Abstract

Internal ribosome entry site (IRES)-mediated translation is a specialized mode of protein synthesis which malignant cells depend on to survive adverse microenvironmental conditions. Our lab recently reported the identification of a group of compounds which selectively interfere with IRES-mediated translation, completely blocking de novo IGF1R synthesis, and differentially modulating synthesis of the two c-Myc isoforms. Here, we examine the phenotypic consequences of sustained IRES inhibition in human triple-negative breast carcinoma and glioblastoma cells. A sudden loss of viability affects the entire tumor cell population after ∼72-h continuous exposure to the lead compound. The extraordinarily steep dose-response relationship (Hill-Slope coefficients −15 to −35) and extensive physical connections established between the cells indicate that the cells respond to IRES inhibition collectively as a population rather than as individual cells. Prior to death, the treated cells exhibit prominent features of terminal differentiation, with marked gains in cytoskeletal organization, planar polarity, and formation of tight junctions or neuronal processes. In addition to IGF1R and Myc, specific changes in connexin 43, BiP, CHOP, p21, and p27 also correlate with phenotypic outcome. This unusual mode of tumor cell death is absolutely dependent on exceeding a critical threshold in cell density, suggesting that a quorum-sensing mechanism may be operative. Death of putative tumor stem cells visualized in situ helps to explain the inability of tumor cells to recover and repopulate once the compound is removed. Together, these findings support the concept that IRES-mediated translation is of fundamental importance to maintenance of the undifferentiated phenotype and survival of undifferentiated malignant cells.

**Electronic supplementary material:**

The online version of this article (doi:10.1007/s13277-016-5161-4) contains supplementary material, which is available to authorized users.

## Introduction

General protein synthesis is accomplished by ribosomal scanning from the 5′ end of the messenger RNA (mRNA), while internal ribosome entry site (IRES)-mediated translation serves as a fail-safe or emergency system-driving synthesis of proteins critical for survival, especially under adverse conditions when general protein synthesis is compromised [1–5]. Gene-specific translation-regulatory mechanisms such as the IRES play an extremely important role in determining gene product levels, and consequently, the phenotype of the cell [6–10]. It is estimated that 10–15 % of all human genes may be translated via an IRES, but only a small fraction of these have been identified and characterized [11]. Accumulating evidence suggests that malignant cells may be particularly dependent upon IRES-mediated translation, and may exploit this mechanism to synthesize certain growth factor receptors, proto-oncogenes, and apoptosis-regulatory proteins to promote their own survival [12–15]. We and others have shown that IRES-mediated translation of oncogenic mRNAs such as *c-myc* and *IGF1R* may be responsible for, or contribute to resistance to therapy and enhanced survival of malignant cells under suboptimal microenvironmental conditions such as those to which tumor cells are exposed in vivo [16–18].

Our lab has sought to develop chemical probes capable of selectively modulating IRES function. We recently reported the identification of a group of compounds (prototype IRES inhibitors) for which mechanism of action was confirmed, and effects on the *IGF1R* and c-*myc* IRESs were examined in detail [19]. The identification of these compounds allows us to selectively perturb IRES-mediated translation in its native context, and investigate its relationship to the malignant phenotype. Here, we focus on the phenotypic consequences of IRES inhibition, characterizing the atypical mode of cell death triggered following continuous exposure to the lead compound.

The experiments utilize two human tumor models, triple-negative breast carcinoma and glioblastoma, both of which are highly undifferentiated, and for which new treatment approaches are needed to address major inadequacies in our current therapeutic armamentarium. The results point toward an integral relationship between IRES-mediated translation and the undifferentiated state, demonstrating that chemical interference with IRES function is capable of inducing a phenotypic shift closely resembling terminal differentiation, which is followed closely by loss of viability affecting the entire tumor cell population.

## Materials and methods

### Cells and cell culture

SUM159PT human breast carcinoma cells, which are triple-negative (negative for estrogen receptor α, progesterone receptor, and non-amplified Her2), were obtained from Asterand and propagated in Ham’s F-12 media supplemented with 5 % fetal calf serum (FCS), 10 mM HEPES, 5 μg/ml insulin, and 1 μg/ml hydrocortisone. T98G human glioblastoma cells were obtained from ATCC and propagated in MEM supplemented with 10 % FCS, 1 % non-essential amino acids, and 10 μg/ml insulin. Normal primary human mammary epithelial cells (HMEC, derived from reduction mammoplasty) were obtained from Lonza and propagated in mammary epithelial basal medium supplemented with bovine pituitary extract, EGF, insulin, and hydrocortisone as recommended by the supplier. 143B osteosarcoma cells were obtained from ATCC and propagated in EMEM supplemented with 10 % FCS. Except when deliberately varied, experiments were set up with cells seeded at 22.5–30 % density relative to confluence (∼60–80,000 cells/cm^2^). Low serum conditions (0.5 % FCS, no supplemental insulin) were frequently used to assess the degree to which dependence on IRES-mediated translation and sensitivity to IRES inhibition are enhanced when the microenvironment to which cells are exposed is suboptimal, e.g., limiting soluble growth factors. HMECs were subjected to low growth factor conditions by diluting full propagation media 1:9 with unsupplemented basal media.

### Reagents and antibodies

IRES inhibitor lead compound P (cpd_P) = N-(4-anilinophenyl)-N′-[2-(4-chlorophenyl)ethyl]thiourea, MW 381, [19] was solubilized in 100 % dimethyl sulfoxide (DMSO) to a concentration of 10 mg/ml and used immediately or stored at −20 °C. Stock solutions were diluted a minimum of 1:500 in media such that final DMSO concentration did not exceed 0.2 %, which was matched in vehicle-only control samples. Compounds were thoroughly dispersed in media before adding to cells and incubating for up to 72 h. For incubations extending beyond 72 h, media was changed and compound re-added. For washout/recovery assays, media was replaced (without compound) and cells allowed 24–72 h to resume proliferation.

Antibodies: c-Myc (N262, rabbit, Santa Cruz); IGF1R (C-20, rabbit, Santa Cruz); BiP (clone 40, mouse, BD); CHOP (L63F7, mouse, Cell Signaling); ZO-1 (rabbit (Mid), Life Technologies, 1:100 for imaging); β-III-tubulin (TUJ1, mouse, Covance, 1:500 for imaging); connexin 43 (rabbit, Cell Signaling); PARP (rabbit, Cell Signaling); cleaved caspase 7 (Asp198, D6H1, rabbit monoclonal, Cell Signaling); pRb (4H1, mouse, Cell Signaling); cyclin D1 (H-295, rabbit, Santa Cruz); E2F1 (C-20, rabbit, Santa Cruz); p21 (F-5, mouse, Santa Cruz); p27 (clone 57, mouse, BD); and α-tubulin (B-5-1-2, mouse, Sigma, 1:500 for imaging). Secondary antibodies for indirect immunofluorescence staining were AlexaFluor 488 or 594-conjugated goat anti-mouse or rabbit IgG (highly cross-adsorbed, Life Technologies). Actin was visualized using Alexafluor 488-conjugated phalloidin. DAPI (4′,6-Diamidino-2-phenylindole dihydrochloride) was from Sigma.

### Cell viability assays

Viability endpoints were measured using standard methodologies, and readouts obtained by bioluminescence for ATP (CellTiter-Glo, Promega), cytoplasmic protease (CytoTox-Glo, Promega), or spectrophotometrically for MTT. Cellular ADP was measured sequentially with ATP, following addition of an excess of enzyme capable of converting ADP to ATP (Abcam). Protease activity (endogenous aminopeptidase cleaving the Ala-Ala-Phe-aminoluciferin substrate [20]) was assayed separately in aliquots of media (as a measure of cell death) as well as lysates prepared from the adherent cells (as a measure of cell viability). Appropriate negative controls (lysis buffer alone, media alone) were subtracted from all readings.

### Dose-response analyses

Viability data were analyzed and Hill slope coefficients calculated by non-linear regression using the dose response-inhibition-variable slope (four parameters) module of GraphPad Prism 6.

### Western blot assays

Whole cell lysates were prepared from treated cells as described [19] using lysis buffer containing 4 % SDS and 720 mM 2-mercaptoethanol, preheated to 100 °C. Equivalent aliquots of protein from each sample were separated by SDS/PAGE, transferred to 0.2 μm nitrocellulose membrane, and probed with antibodies using standard immunoblotting procedures.

### Indirect immunofluorescence staining

Cells were seeded in 8-well chamber slides (Nunc) and allowed 48 h to recover and resume proliferation prior to treatment with cpd_P or vehicle control (0.1 % DMSO) as indicated in individual figure legends.

Cells were fixed with freshly prepared 2 % paraformaldehyde X 15 min at room temperature, followed by permeabilization with 0.2 % Triton X-100 X 10 min. After washing in PBS with 75 mM glycine and blocking in 5 % normal goat serum, primary antibody was added and incubated for 45 min to 1 h at room temperature. Following two washes in PBS and a 10-min re-blocking step, secondary antibodies (goat anti-rabbit IgG or anti-mouse IgG, highly cross-adsorbed, conjugated to AlexaFluor 488 or 594, 1:200) were added and incubated for 35–45 min. Cells were washed twice, nuclei stained with DAPI (0.2 μg/ml), and mounted using ProLong Gold (Life Technologies).

### Confocal imaging

Images were captured using a Nikon A1 confocal instrument with 40X 1.3NA objective. In most cases, fields were randomly selected for imaging on the basis of DAPI staining pattern alone (the exception being PKH26 staining of candidate tumor stem cells, for which dedicated search for fields containing bright red cells was necessary). Paired images of control and experimental wells were acquired sequentially and all settings including laser power, PMT voltage, and pinhole were held constant between samples.

### PKH26 staining

Initial staining of stock cultured cells was performed as recommended by the supplier (Sigma). Briefly, ∼4 × 10^6^ freshly harvested cells were incubated (in suspension) with 2 μM PKH26 in 500 μl of Diluent C for 4 min at room temperature. Excess dye was scavenged by addition of equal volume FCS for 1 min, followed by three washes in 10 ml serum-containing media, after which the cells were returned to culture. Following 7 days for propagation and mitotic dilution of dye, cells were harvested and utilized to set up experiments.

### Live cell imaging

Cells were seeded in 8-well chambered coverslips (Nunc, 400 μl / well) and treated with cpd_P for appropriate periods of time as indicated on each figure. Subsequent addition of other compounds (Z-VAD-fmk, necrostatin) and staining reagents (CMXRos, Hoescht 33258) was accomplished without changing existing media, by adding diluted reagent to 200 μl aliquot of existing media (temporarily withdrawn from well), mixing, and then adding back to the original well. CMXRos (chloromethyl-X-rosamine, final concentration 100 nM) and Hoescht 33258 (final concentration 2 μg/ml) were added 1 h prior to imaging. Calcein acetoxymethyl (AM) was freshly dissolved in DMSO for a 4 mg/ml stock solution, an intermediate dilution of 40 μg/ml prepared in media, and 5 μl of this added directly to well 5 min prior to imaging (final concentration 0.5 μg/ml).

Calcein AM serves as an indicator of cell viability. While the acetoxymethyl (AM) ester of calcein is not fluorescent, esterases present within the cytoplasm of viable cells readily cleave the AM moiety, releasing free calcein which is fluorescent, producing bright green images. Chromatin condensation accompanying cell death is associated with intense Hoescht 33258 (blue) staining of the nucleus.

## Results

### Prolonged exposure to IRES inhibitor cpd_P, beyond critical thresholds in concentration and time, results in massive loss of viability of triple-negative breast tumor cells, with no evidence for recovery following extended incubation in absence of the compound

**Fig. 1:**
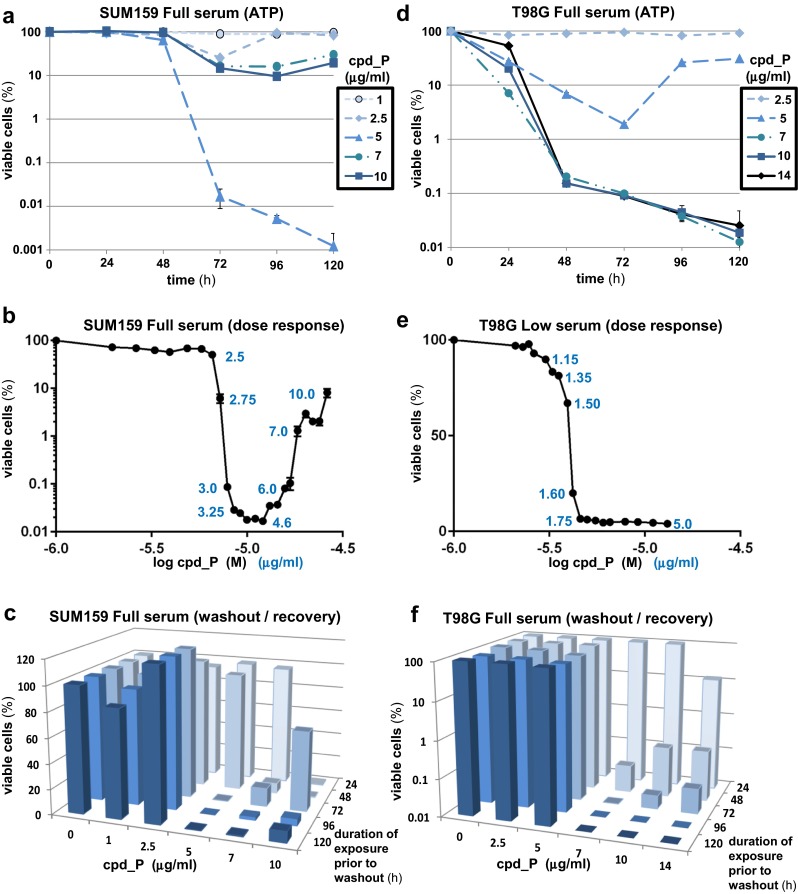
Sustained IRES inhibition results in massive loss of viability in triple-negative breast carcinoma and glioblastoma cells. **a** SUM159 breast tumor cells were treated with increasing concentrations (0–10 μg/ml) of IRES inhibitor cpd_P for periods of 24 to 120 h. Cell survival is assessed using ATP (CellTiter-Glo) as endpoint, presented relative to vehicle (0.1 % DMSO) control (=100 %), plotted on a logarithmic scale. All data ± SEM. **b** Tight dose-response titration for SUM159 cells treated with cpd_P in full serum. Assay endpoint is ATP at 72 h. Shown are the actual data ± SEM in triplicate. The most comprehensive tumor cell death is obtained at intermediate concentrations of cpd_P between 3 to 6 μg/ml. As the cpd_P concentration approaches this intermediate range, an extremely sharp decline in cell viability is evident (note near 1000 fold decrease in viability between 2.5 and 3.0 μg/ml cpd_P). Non-linear regression was used to fit the data to standard 4-parameter (variable slope) dose-response equation. Hill Slope Coefficient = −35.09 (95 % confidence interval −37.63 to −32.54), *R* square = 0.9987. **c** Washout/recovery assay measuring capacity of SUM159 cells to repopulate following exposure to IRES inhibitor. Cells were treated with cpd_P at variable concentrations (range 0–10 μg/ml) for variable periods of time (range 24–120 h). Compound was then removed and cells allowed 72 h to recover and resume proliferation. **d** T98G glioblastoma cells were treated with increasing concentrations (0–14 μg/ml) of cpd_P (or vehicle control = 0.14 % DMSO) for varying periods of time as indicated. Cell survival is assessed using ATP as endpoint (logarithmic scale). All data ± SEM. **(e)** Tight dose-response titration for T98G cells treated with cpd_P under Low serum conditions (0.5 % FCS, no supplemental insulin), over the range 0.8 to 5 μg/ml. Assay endpoint is MTT at 72 h. The data were analyzed by non-linear regression to fit the standard 4-parameter (variable slope) dose-response equation. Hill Slope coefficient = −15.51 (95 % confidence interval = −20.79 to −10.22), R square = 0.9811. **f** Washout/recovery assay for T98G cells treated with increasing concentrations of cpd_P (range 0–14 μg/ml) for varying periods of time (range 24 to 120 h). Following treatment, compound was removed and cells allowed an additional 72 h to recover and resume proliferation. Endpoint is ATP, plotted on logarithmic scale. Note the sharp thresholds in concentration and duration of exposure required for irreversible elimination of the tumor cells

We hypothesized that selective interference with IRES function would be detrimental to tumor cells which depend on IRES-mediated translation of key oncogenic proteins such as IGF1R and Myc for their survival. Using the human triple-negative breast carcinoma cell line SUM159 as our initial model, we observe that continuous exposure to IRES inhibitor lead compound P (cpd_P) results in significant loss of viability (Fig. 1a, b, Suppl. Fig. 1a). Three aspects of the viability titration are particularly notable. First, cell death is sub-acute in nature, requiring ∼72-h continuous exposure to the IRES inhibitor. Second, it is possible to approach complete obliteration of the tumor cell population, eliminating all or very nearly all of the tumor cells (<0.01 % cell survival). Third, the most effective elimination of tumor cells occurs not at the highest concentration, but at intermediate concentrations of cpd_P (∼5 μg/ml under full serum conditions). A further increase in cpd_P concentration (≥7 μg/ml) is actually less effective in terms of tumor cell eradication.

This pattern of sensitivity to cpd_P was confirmed using a cytoplasmic protease (Promega) as an alternative endpoint for assessing cell survival (Suppl. Fig. 1b). A perfectly reciprocal pattern was obtained by measuring protease activity released into the media (an indication of loss of cell membrane integrity expected to accompany cell death). In fact, three distinct measures of cell viability: ATP, cytoplasmic protease, and MTT (as previously reported [19]) are perfectly concordant in identifying the intermediate concentration of cpd_P as the most effective (Suppl. Fig. 1c). The three measures of viability are also similar in terms of temporal profiles (Suppl. Fig. 1d), although release of cytoplasmic protease into the media clearly lags behind the decline in ATP or MTT metabolic activity, suggesting that loss of cell membrane integrity may be delayed in this particular mode of cell death. No significant elevation of ADP levels or ADP/ATP ratio is observed (Suppl. Fig. 1e), suggesting that this mode of cell death is not likely to involve necrosis, and may be distinct from classical apoptosis as well (both of which are associated with increased ADP/ATP [21]). In fact, there is actually a decrease in ADP/ATP ratio at 48 h with higher concentrations of cpd_P.

Non-linear regression of the cpd_P titration (fitting the curve to the canonical dose-response equation) reveals a Hill slope coefficient of −35 (in contrast to a typical Hill slope range of −0.4 to −4.0 associated with many anti-cancer drugs currently in use [22, 23]). The slope of the dose-response curve is indicative of the degree of homogeneity in response, with a steep curve indicative of a more uniform response across the population [23]. Thus, the extraordinarily steep slope characterizing the cpd_P titration indicates that essentially the entire population of breast tumor cells is responding in a similar manner and in the same time frame.

Washout/recovery assays were performed to test the capacity of the tumor cells to rebound and repopulate following cessation of treatment, and examine more precisely the point at which irreversible commitment to cell death has taken place (Fig. 1c). Very sharp thresholds in both concentration and duration of exposure are observed. The results indicate that, at the optimal intermediate concentration of cpd_P (5 μg/ml), loss of viability initiates suddenly between 48 and 72 h, and impacts essentially the entire breast tumor cell population within that 24-h period. Cells treated with cpd_P when either concentration or duration of exposure is below threshold display robust recovery upon removal of the compound, indicating that transient or suboptimal IRES inhibition is reversible and does not appear to compromise significantly the replicative capacity of the tumor cells. However, cells treated beyond these critical thresholds of both concentration and time exhibit no evidence for recovery even following prolonged incubation in absence of the compound.

Results obtained under low serum conditions (0.5 % FCS, no supplemental insulin) illustrate how the stress imposed by limiting growth/survival factors increases reliance on IRES-mediated translation and sensitivity to IRES inhibition (Suppl. Fig. 1f). The dose-response shifts significantly (3–5-fold) to the left, such that a cpd_P concentration of ∼1 μg/ml is now optimal for tumor cell death. Two structural analogs of cpd_P (P-2, P-3 [19]) were also tested, and precisely recapitulate the biphasic pattern of response seen with the parent compound (Suppl. Fig. 1g).

### Glioblastoma cells display a similar pattern of susceptibility to IRES inhibition

Human glioblastoma cells (T98G) respond to IRES inhibition in almost precisely the same manner as the triple-negative breast carcinoma cells (Fig. 1d). The titrations are marked by a sharp threshold in concentration of cpd_P (∼7 μg/ml) required to eliminate the tumor cells (∼99.9 % cell death by 48 h and approaching 4-log cell kill by 120 h). Loss of cell membrane integrity and release of cytoplasmic contents (e.g., protease) are significantly delayed in comparison to the other endpoints (Suppl. Fig. 2a). A similar pattern is observed under low serum conditions (Fig. 1e), although with a significant shift of the curve to the left, indicative of a substantial increase in sensitivity to the IRES inhibitor. Again, the dose-response curve is extraordinarily steep (Hill slope coefficient of −15.5, 95 % confidence interval −10.2 to −20.7), suggesting that cell death at the optimal concentration of cpd_P is a consequence of a specific phenotypic alteration brought about by the inhibition of synthesis of critical IRES-driven proteins, and not a typical cytotoxic response. Analogs P-2 and P-3 exhibit the same steep dose-response profile as the parent compound (Suppl. Fig. 1h). Not only do the tumor cells fail to recover and repopulate once compound is removed (Fig. 1f), it appears that viability of the small fraction of remaining cells is even further compromised during the washout/recovery period, falling as much as an additional two logs (approaching the limit of resolution for the scale of the experiment, Suppl. Fig. 2b).

### Normal cells are resistant to IRES inhibition

Although it may be pathologically activated in undifferentiated tumor cells, under normal physiological conditions, the c-*myc* IRES is utilized primarily during early embryonic development, and is essentially inactive in normal differentiated adult tissues [12]. Consequently, it is reasonable to postulate that normal cells might not be reliant on IRES-mediated translation, and might not be adversely affected by prolonged exposure to an IRES inhibitor. To test this, normal primary human mammary epithelial cells (HMECs) were treated with IRES inhibitor cpd_P for 72 h under either full (growth factor supplemented) or low (growth factor deprived) conditions. Little or no loss of viability of the normal cells was observed (Suppl. Fig. 2c, d). In contrast, malignant osteosarcoma cells (143B) display a pattern of sensitivity to IRES inhibition that closely resembles that of the SUM159 breast carcinoma and T98G glioblastoma cells (Suppl. Fig. 2e, f). Thus, it appears that while IRES inhibition can be highly detrimental to undifferentiated tumor cells which depend on IRES-mediated translation for their survival, normal cells may tolerate such a treatment.

### IRES inhibition causes cells to display features of terminal differentiation prior to synchronized loss of viability

**Fig. 2:**
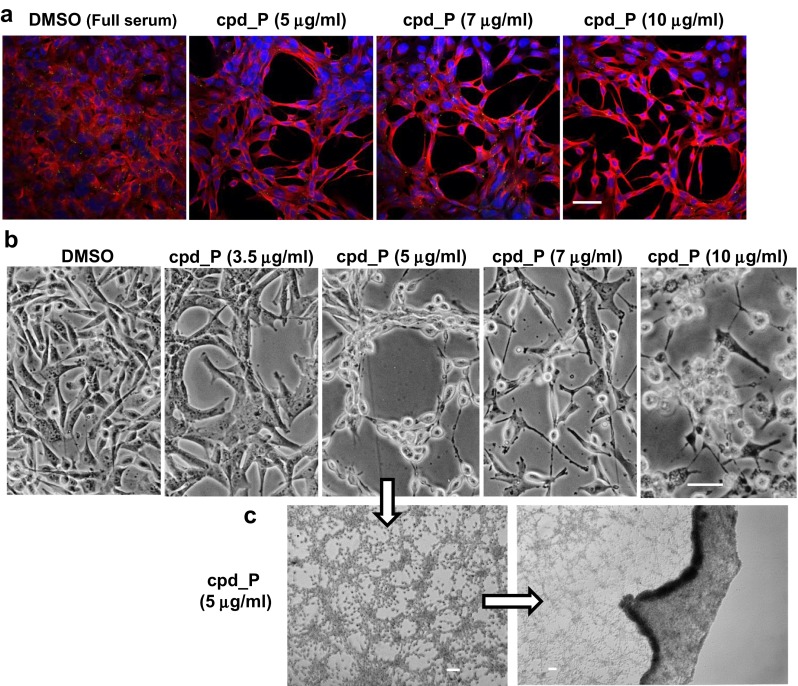
IRES inhibition induces marked gains in structural organization and intercellular networking of breast tumor cells. **a** α-tubulin staining (*red*) and confocal imaging of SUM159 breast tumor cells treated under full serum conditions for 36 h with IRES inhibitor cpd_P at the indicated concentrations. **b** Phase contrast images of SUM159 cells continuously exposed to cpd_P at concentrations ranging from 0 to 10 μg/ml for 72 h under full serum conditions. Note the highest degree of structural organization and most comprehensive cell death is obtained at intermediate concentration of cpd_P (5 μg/ml). Also note the homogeneous morphology at 5 μg/ml versus the bimorphic appearance of cells treated at 7–10 μg/ml cpd_P. **c** Phase contrast images of SUM159 cells treated with 5 μg/ml cpd_P for 60 h under full serum conditions, demonstrating extensive intercellular connections formed in response to IRES inhibition. The image on the *right* was obtained at low magnification and demonstrates the tendency of this network of dead cell corpses to peel off the growth surface as an intact sheet. *Scale bars* 50 μm (**a**, **b**); 100 μm (**c**)

SUM159 triple-negative breast tumor cells early in the course of treatment with IRES inhibitor cpd_P exhibit dramatic alterations in structural organization and spatial orientation (Fig. 2a, Suppl. Fig. 3). The α-tubulin staining pattern is indicative of a more highly developed cytoskeleton. The cells also demonstrate a propensity to form distinct circular structures, with remarkably well-rounded concave surfaces surrounding open spaces (evidence of planar polarity), and form extensive connections with neighboring cells. By contrast, the vehicle-treated cells exhibit only diffuse cytoplasmic α-tubulin staining and remain haphazardly distributed on the growth surface, with little or no evidence of intercellular interaction or polarity.

As cells approach 72-h continuous exposure to cpd_P at the optimal concentration of 5 μg/ml, a profound loss of cytoplasmic volume is observed accompanying loss of viability, yet the nucleus retains its round contour, and the intercellular connections between the cell corpses remain intact (Fig. 2b, middle panel). The end result is a vast network of interconnected circular structures resembling a chain-link fence, covering the entire growth surface (Fig. 2c). The homogeneous appearance of the cells correlates with the comprehensive loss of viability as documented in Fig. 1. The cells do not detach individually from the growth surface as is typical of apoptosis or programmed necrosis, but remain attached. Eventually, the network of interconnected dead cell corpses peels off as an intact sheet, in a manner reminiscent of desquamation. This helps to explain the delay in release of cytoplasmic contents (e.g., protease) noted earlier, in that loss of cell membrane integrity and cellular disintegration are not key features of this mode of cell death.

Note that the highest degree of structural organization and intercellular connectivity is observed at the intermediate concentration of cpd_P (5 μg/ml in full serum). Cells treated with lower concentrations of cpd_P show signs of increased cellular interdependence and structural organization, but on a considerably more limited scale, and with little or no loss of viability. Cells treated with higher concentrations of cpd_P (e.g., 7–10 μg/ml) beyond the window for optimal cell death are less highly-organized and loss of viability, while extensive, is not comprehensive, as a subpopulation of relatively resistant or at least more slowly dying cells can readily be seen. These findings are consistent with the biphasic viability data presented in Fig. 1.

### Glioblastoma cells subjected to IRES inhibition also exhibit marked gains in structural organization and formation of an extensive intercellular network

**Fig. 3:**
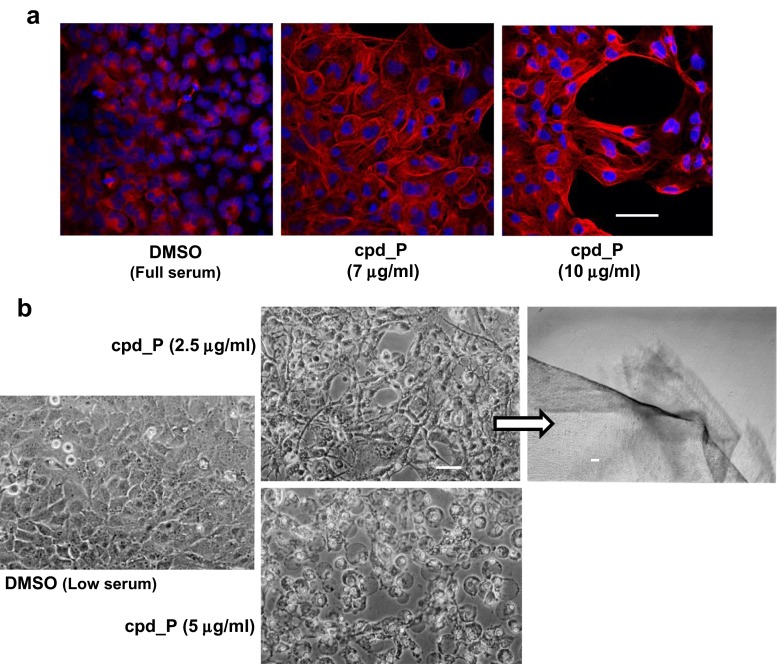
IRES inhibition induces marked gains in structural organization and intercellular networking of glioblastoma cells. **a** α-tubulin (*red*) staining patterns in T98G glioblastoma cells treated under full serum conditions for 48 h with IRES inhibitor cpd_P at the indicated concentrations. **b** Phase contrast images of T98G cells exposed to cpd_P for 48 h under low serum (0.5 % FCS) conditions. Note the distinct phenotypic outcomes associated with the intermediate (2.5 μg/ml) versus high (5 μg/ml) concentrations of cpd_P. The image in the *upper right panel* was obtained at low magnification of cells treated with 2.5 μg/ml cpd_P, and demonstrates the tendency of these cells to peel off the growth surface as an intact sheet. *Scale bars* 50 μm (except for **b**) *upper right* = 100 μm

Perhaps surprisingly, the morphological changes observed in T98G glioblastoma cells treated with IRES inhibitor cpd_P closely resemble those seen in the triple-negative breast tumor cells. Confocal imaging of α-tubulin staining patterns again documents a marked gain in cytoskeletal organization both within the individual cells as well as overall tissue architecture (Fig. 3a). When treated with optimal (intermediate) concentrations of cpd_P (e.g., 2.5 μg/ml in low serum), many of the glioblastoma cells adopt a wiry appearance, developing long processes, suggestive of neuronal differentiation (Fig. 3b). The cells display increased intercellular connectivity, forming a tight network that, much like that formed by the breast tumor cells, remains attached to the growth surface, eventually peeling off as an intact layer.

Although the quantitative distinction in cell death outcomes between intermediate and higher concentrations of cpd_P is not as marked in the glioblastoma cells as with the breast tumor cells (compare Fig. 1b to Fig. 1e), the morphological distinction between these phenotypic outcomes is startling (Fig. 3b, middle panels), as cells treated with cpd_P at concentrations beyond the optimal window for differentiation exhibit gross osmotic swelling and widespread detachment of individual cells.

### Critical threshold in cell density required for triggering of synchronized, comprehensive tumor cell death

The fact that the mode of cell death induced by intermediate concentrations of IRES inhibitor cpd_P appeared to be synchronized and comprehensive in nature, with essentially all of the cells undergoing a dramatic phenotypic shift characterized by features of terminal differentiation, followed by massive loss of viability, suggested that this was a highly orchestrated event, in which the population of malignant cells responded as a unit. Furthermore, the extensive intercellular connections established (so structurally sound as to be retained even following cell death) suggested that communication and interaction among the population of cells might be a key to executing this phenotypic alteration and the subsequent loss of viability.

**Fig. 4:**
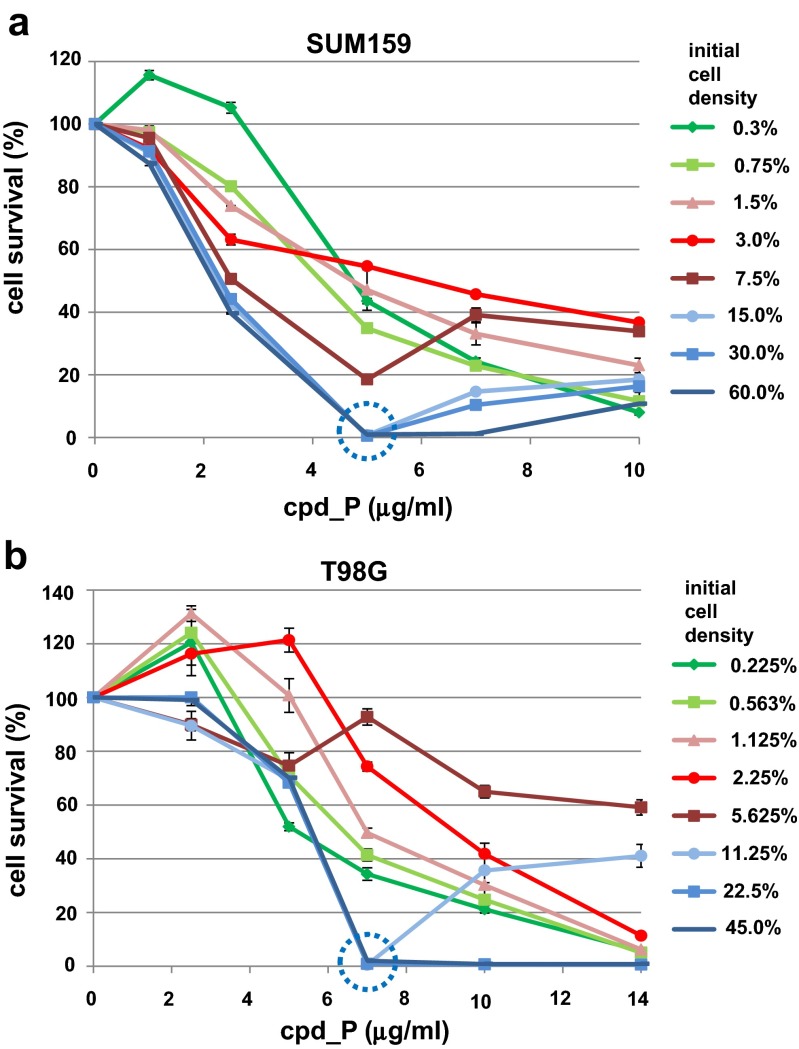
Titration of cell density and its effect on susceptibility to IRES inhibition. SUM159 breast tumor cells **(a)** or T98G glioblastoma cells **(b)** were seeded at varying densities, allowed 48 h to recover and resume proliferation, then treated with IRES inhibitor cpd_P at varying concentrations for 72 h. Relative cell survival is plotted versus cpd_P concentration, where each line represents a different initial seeding density. *Blue* above threshold density, conducive to terminal differentiation and susceptible to synchronized, comprehensive cell death triggered by IRES inhibition; *red* below threshold, resistant to intermediate concentrations of cpd_P; *green* very low (clonogenic) density. All data ± SEM

To test this hypothesis, a series of experiments was performed in which cell death outcome in response to IRES inhibition was titrated as a function of cell density. The results obtained for both SUM159 breast tumor cells (Fig. 4a, Suppl. Fig. 4a) and T98G glioblastoma cells (Fig. 4b, Suppl. Fig. 4b) clearly indicate that there exists a very sharp threshold in cell density which is required in order for the synchronized, comprehensive mode of cell death to take place. SUM159 cells seeded at ≥15 % (∼40,000 cells/cm^2^) precisely recapitulate the biphasic viability patterns described in Figs. 1 and 2, with essentially complete elimination of the population (≥99 % cell death) at the optimal cpd_P concentration of 5 μg/ml. However, cells seeded at just half that density (7.5 %) are affected but not comprehensively eliminated, and cells seeded at 3 % are essentially resistant to cell killing by cpd_P. A similar relationship between cell density and susceptibility to IRES inhibition is observed in the T98G glioblastoma cells, where cells seeded at 11.25 % (∼45,000 cells/cm^2^) are almost completely eliminated (0.6 % survival) following 72-h exposure to 7 μg/ml cpd_P, but cells seeded at half that density (5.625 %) are completely resistant (92.8 % survival) to the same treatment. These results indicate that the unique mode of cell death observed at intermediate concentrations of cpd_P is *a population event*.

This requirement for a minimum number or density of cells is consistent with the physiological purpose of terminal differentiation, a program executed by a population of cells intended to form a functional tissue. It is a fundamental principle of development that terminal differentiation should normally not be initiated until a sufficient number of progenitor cells have accumulated to form the intended tissue or organ. This apparent monitoring of cell number or population density by the tumor cells is highly reminiscent of the quorum-sensing mechanisms utilized by bacteria to coordinate population-wide changes in phenotype. Quorum sensing is a form of intercellular communication which allows unicellular prokaryotic organisms to respond synchronously to alterations in microenvironment, almost as if they were a multicellular organism [24, 25]. The involvement of quorum-sensing mechanisms in human tumor cell biology and metastasis has been hypothesized [26, 27].

### Further evidence of terminal differentiation induced by IRES inhibition: formation of tight junctions and neuronal processes

**Fig. 5:**
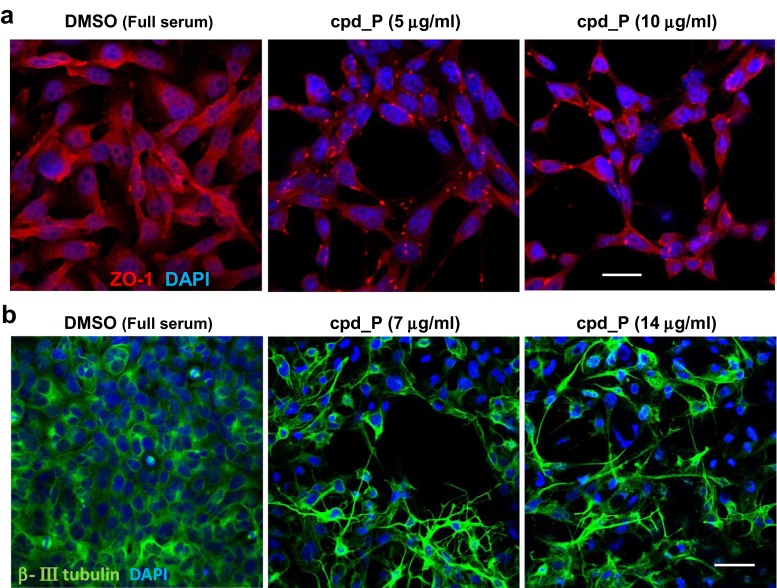
Evidence of terminal differentiation in breast tumor and glioblastoma cells subjected to IRES inhibition: formation of tight junctions and neuronal processes. **a** SUM159 breast tumor cells treated with cpd_P at 5 or 10 μg/ml for 48 h under full serum conditions, then stained for ZO-1 (Tight Junction Protein 1, *red*). Focal distribution of ZO-1 in treated cells is consistent with formation of tight junctions. **b** T98G glioblastoma cells treated with cpd_P at 7 or 14 μg/ml for 48 h under full serum conditions, then stained for β-III tubulin (a marker of neuronal differentiation, *green*). *Scale bar* = 25 μm (**a**); 50 μm (**b**)

In vehicle-treated SUM159 cells, low-level ZO-1 immunoreactivity is spread diffusely throughout the cytoplasm; however, in cells exposed to cpd_P, brightly stained discrete ZO-1-containing foci are present at points of intercellular contact, suggestive of the formation of functional tight junctions, a feature of differentiated epithelia (Fig. 5a, Suppl. Fig. 5). Interestingly, cells treated with cpd_P at the optimal concentration of 5 μg/ml display multiple ZO-1 positive foci per cell and make multiple contacts with adjacent cells in the context of round acinar structures. The cells exposed to higher concentrations of cpd_P only display 1 or 2 ZO-1 foci per cell, and the intercellular connections are correspondingly more restricted in these cells.

The wiry morphology and marked gain in intercellular connectivity observed in T98G cells treated with cpd_P is made particularly evident upon staining for β-III tubulin, a specific marker of neuronal differentiation (Fig. 5b). Vehicle-treated control cells are roughly trapezoidal in shape; however, when treated with cpd_P, the cells develop very long processes typical of differentiated neurons. Some of these processes are particularly large in diameter, while others display a branching morphology. Together, these findings support the conclusion that IRES inhibition has the potential to induce a dramatic phenotypic alteration consistent with terminal differentiation, even in what originally were highly undifferentiated malignant cells.

### Specific changes in Myc, IGF1R, BiP, CHOP, and connexin 43 correlate with phenotypic outcome of IRES inhibition

**Fig. 6:**
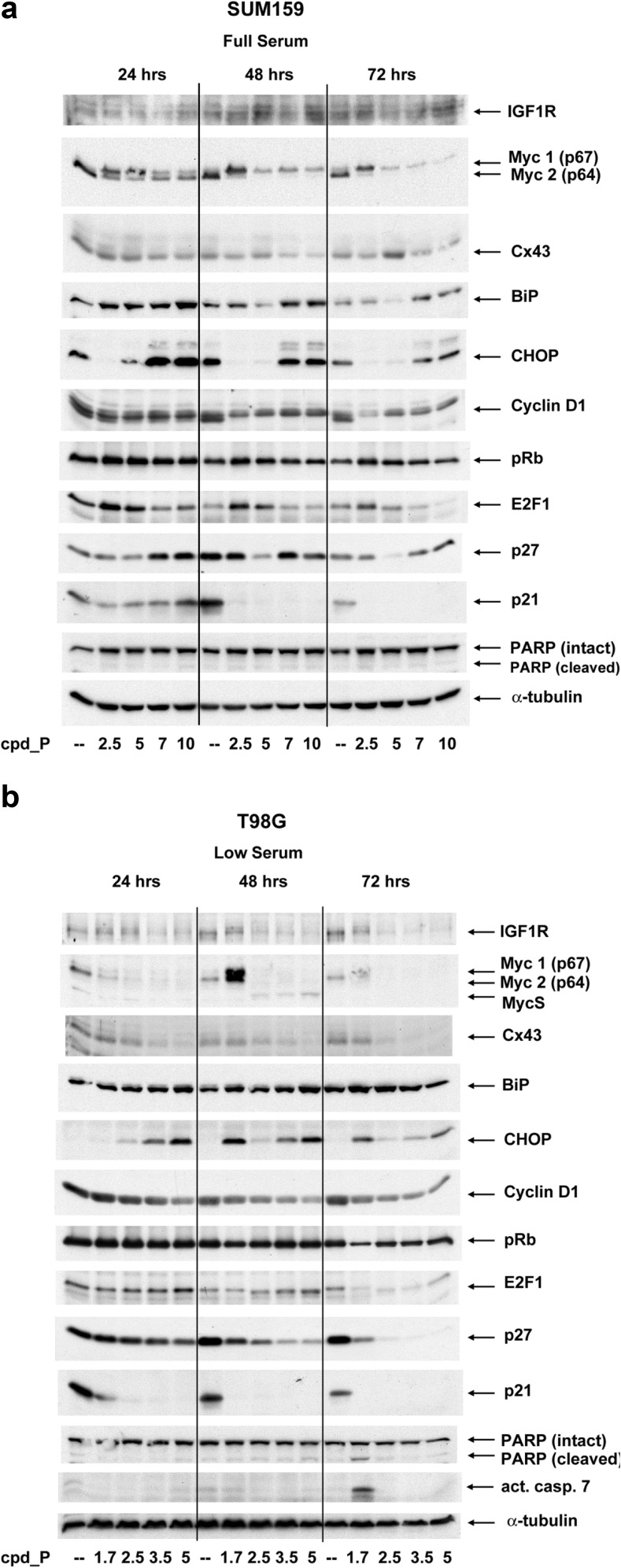
Molecular correlates of tumor cell death induced by IRES inhibition in SUM159 breast tumor cells and T98G glioblastoma cells. **a** Composite western blot results obtained from whole cell lysates prepared from SUM159 cells treated with varying concentrations of cpd_P for 24, 48, or 72 h under full serum (5 % FCS, 5 μg/ml insulin) conditions. Myc 2 (p64) is the dominant (oncogenic) isoform of c-Myc. Myc 1 (p67) is the minor (growth inhibitory) isoform of c-Myc. **b** Composite western blot results obtained from whole cell lysates prepared from T98G cells treated with varying concentrations of cpd_P for 24, 48, or 72 h under low serum (0.5 % FCS, no supplemental insulin) conditions. MycS is generated by translation initiation at either of two closely spaced internal methionine codons within exon 2 [28]

Western blot analyses reveal dynamic alterations in key molecules which help to explain the outcomes of IRES inhibition and elucidate the basis for the thresholds and transitions between these outcomes (Fig. 6a).

Dynamic alterations to Myc translation are observed in the SUM159 breast tumor cells, with a shift from synthesis of the dominant oncogenic isoform p64 to the minor isoform p67, which has been attributed potent growth inhibitory properties [29]. The most complete shift to p67 occurs at the optimal concentration of 5 μg/ml cpd_P. By 48 h, cells treated across the range of 5–10 μg/ml cpd_P exhibit exclusively the p67 isoform, and at 72 h, even the level of p67 diminishes such that very little Myc protein remains in these cells. The pattern observed under low serum conditions is similar (Suppl. Fig. 6a).

For T98G, essentially all Myc protein is lost from the cpd_P-treated cells (Fig. 6b, Suppl. Fig. 6b). Under low serum conditions, the cells display evidence of attempts to counteract the perpetual interference with IRES-mediated translation of Myc. At 1.75 μg/ml cpd_P, Myc is transiently superinduced, perhaps by shifting c-*myc* transcription to use of a different mRNA isoform. Cells treated with ≥2.5 μg/ml cpd_P exhibit low level synthesis of MycS, a naturally-occurring N-terminally truncated isoform generated by translation initiation at either of two downstream AUG codons within exon 2 [28]. Apparently, neither of these attempts to circumvent the block to IRES-mediated translation of Myc is sufficient to salvage the cells, as a 4 to 5 log cell kill is observed in each of these situations (Suppl. Fig. 2b).

As Myc is tightly associated with maintenance of the undifferentiated phenotype [30, 31], the change in Myc translation leading to the depletion of Myc protein is almost certainly one of the key factors triggering terminal differentiation and synchronized death in the breast tumor and glioblastoma cells subjected to IRES inhibition.

In SUM159 cells under full serum conditions, there is little turnover of existing IGF1R, and minimal requirement for de novo IGF1R synthesis, thus inhibition of IRES-mediated translation has little or no perceptible impact (Fig. 6a). When serum deprived, however, the SUM159 cells are stimulated to increase synthesis of IGF1R in attempt to more effectively capture limiting soluble ligand (IGF-1 and IGF-2) in the media. This increase in rate of translation of the *IGF1R* mRNA depends on the IRES, and a concentration-dependent effect of cpd_P on IGF1R is revealed (Suppl. Fig. 6a).

IGF1R decreases in a concentration-dependent manner under both full and low serum conditions in T98G cells treated with cpd_P. Interestingly, the optimal cell death outcomes are observed under conditions in which IGF1R is reduced but not eliminated. It is possible that a certain level of IGF1R is required to facilitate induction or execution of the terminal differentiation program [32].

Connexin 43 (cx43, gap junction protein alpha 1) was of interest as a potential contributor (via gap junctional intercellular communication [33, 34]) to the coordinated responses to IRES inhibition exhibited by both the breast tumor and glioblastoma cells. Like *IGF1R* and c-*myc*, the *cx43* mRNA has been shown to contain an IRES [35, 36], and in the T98G cells, the western blot analyses reveal a clear concentration-dependent decrease in cx43 levels. The loss of cx43 at the higher concentrations of cpd_P and the resulting impairment in intercellular communication could conceivably contribute to the inability of these cells to fully execute the population-wide terminal differentiation program, thus leading to death by alternative, less comprehensive (i.e., cell autonomous) mechanisms.

BiP (immunoglobulin binding protein, glucose-regulated protein 78 kDa, GRP78), which is involved in translation quality control [37, 38], is induced by cpd_P in the SUM159 breast tumor cells in roughly a concentration-dependent manner. Curiously, however, there is a de-induction of BiP at the intermediate concentration of cpd_P, in association with the phenotypic shift toward terminal differentiation. A key mediator of cell death triggered by translational stress (i.e., downstream of BiP) is CHOP (CCAAT/enhancer-binding protein homologous protein, DNA damage inducible transcript 3 [39, 40]). Marked CHOP induction is observed at the higher concentrations of cpd_P in both the SUM159 and T98G cells. However, CHOP (much like BiP) is selectively de-induced and nearly eliminated at the intermediate concentrations where cpd_P most effectively induces terminal differentiation and synchronized death (also see Suppl. Fig. 7). It is possible that CHOP expression is deliberately repressed by the cells to facilitate execution of the terminal differentiation program, which may take precedence over responding to translational stress. The high levels of CHOP observed at higher concentrations of cpd_P may be responsible at least in part for substitution of the differentiation-associated population-wide cell death event with a more progressive cell autonomous mode of cell death.

p53 is mutated in both SUM159 and T98G. However, RB1 appears to be intact in each of these cell lines, thus components of the RB1 pathway were examined. In SUM159 cells, dynamic alterations are exhibited in E2F1, first increasing, then decreasing across the titration. Reciprocal changes observed in cyclin D1 may reflect an attempt to compensate for the alterations in E2F1 levels. In T98G cells, E2F1 decreases with increasing concentration of cpd_P, but only in full serum. By 72 h, both E2F1 and cyclin D1 are substantially decreased in the treated cells (in both full and low serum). Overall, however, there does not appear to be a clear consistent relationship between variations in cyclin D1 or E2F1 levels and the cell death outcome elicited by cpd_P.

Perhaps surprisingly, the CDK inhibitors p27 and p21 exhibit marked decreases in the breast tumor and glioblastoma cells subjected to IRES inhibition. In SUM159, p27 (much like CHOP) is diminished most dramatically at intermediate concentrations of cpd_P, in cells undergoing terminal differentiation and synchronized death. In T98G cells in full serum, the decrease in p27 is greatest at intermediate concentrations of cpd_P, while in low serum, p27 decreases in a time and concentration-dependent manner across the entire titration. p21 is completely eliminated from both cell lines following greater than or equal sign 48-h exposure to cpd_P.

Myc inhibits the expression of p21 and p27, so the loss of Myc would be expected to increase rather than decrease these proteins. Furthermore, p27 often increases as cells become quiescent, p21 is expected to increase in response to stress, and both p21 and p27 facilitate terminal differentiation. The fact that neither of these CDK inhibitors increases in the treated cells, rather both exhibit a pronounced decrease in expression, suggests that IRES inhibition disrupts the normal physiological responses expected of these proteins, and the functional incompatibilities generated could contribute to the comprehensive cell death outcome observed in these cells.

Further supporting the conclusion that cell death induced by IRES inhibitor cpd_P is atypical, we observe no activation of caspase 7 (commonly involved in response to translational stress) and no significant PARP cleavage, in either cell line under any treatment condition (except for T98G cells exposed to 1.75 μg/ml cpd_P for 72 h under low serum conditions, which follows the superinduction of Myc). Attempts to detect caspase 3/7 activation by confocal imaging using a fluorescent substrate yielded equivocal results at best (data not shown), suggesting that caspase activation if present is limited. This lack of conventional biochemical indicators of programmed cell death fits with the atypical kinetic and morphological characteristics of death induced by cpd_P.

### Live cell imaging confirms sudden death triggered as a consequence of sustained IRES inhibition

**Fig. 7:**
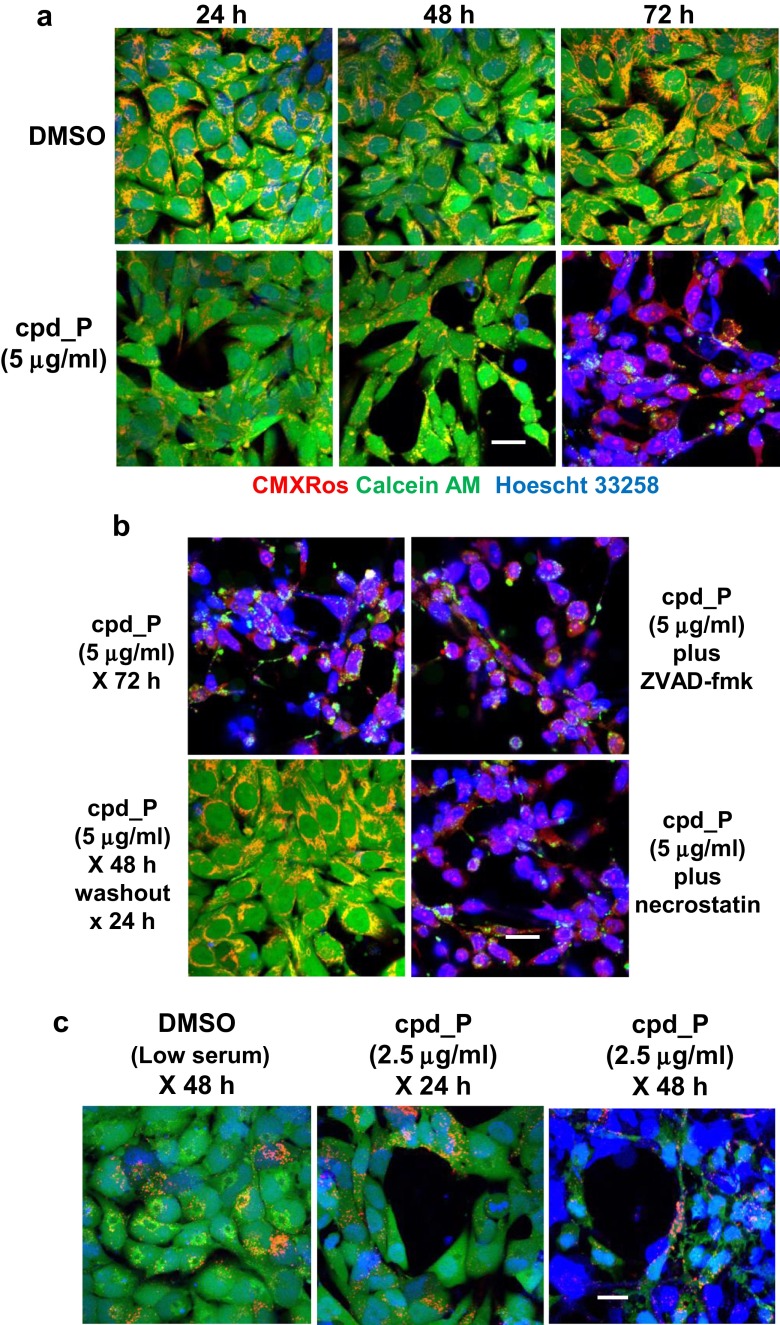
Sudden death triggered by sustained IRES inhibition documented by live imaging. **a** Live cell confocal images of SUM159 cells treated X 24, 48, or 72 h with 5 μg/ml IRES inhibitor cpd_P (or vehicle control) under full serum conditions. *Green* calcein AM; *Red* CMXRos, *Blue* Hoescht 33258. Cells were seeded, treated, stained, and viewed in 8-well chambered coverslips (Nunc). Please see the “Materials and Methods” section for staining protocol. **b** SUM159 cells were treated with cpd_P at 5 μg/ml for 48 h, at which point *i* treatment was continued without alteration; *ii* media was changed and compound removed (washout); or treatment was continued following addition of *iii* Z-VAD-fmk (final concentration 20 μM) or *iv* necrostatin (final concentration 10 μM). Live cell images were captured at the 72-h time point. **c** Live cell confocal images of T98G cells treated with 2.5 μg/ml cpd_P (or vehicle control) for 24 or 48 h under low serum conditions as indicated. All *scale bars* 25 μm

Live cell imaging was carried out on SUM159 cells to enhance our characterization of the atypical mode of cell death associated with IRES inhibition. The cells treated with cpd_P exhibit readily perceptible gains in structural organization and intercellular connectivity within the first 24 h (Fig. 7a), and through 48 h, the cpd_P-treated cells remain viable and actively metabolizing calcein AM (producing green fluorescent signal). Chromatin remains in an open configuration, apparently facilitating execution of the terminal differentiation program.

The images captured at 72 h demonstrate how suddenly and dramatically cell status has changed. The cells are no longer viable, as there is very little calcein AM metabolism, and what little green staining is present is highly dysmorphic and restricted to subcellular pockets of esterase-positive material. Virtually all nuclei now exhibit gross chromatin condensation (manifested as intense Hoescht 33258 (blue) staining). Intercellular connections appear to remain intact, though none of the markers being used for live cell imaging is ideally suited for visualizing the structural components of the network of dead cell corpses.

Figure 7b documents that any adverse consequences of IRES inhibition up to the 48-h time point are reversible, as cells in which the compound was removed at that time rapidly regain the morphology and staining pattern typical of vehicle-treated control cells. In contrast, neither the caspase inhibitor Z-VAD-fmk or the inhibitor of programmed necrosis necrostatin added at the 48-h time point mitigates the population-wide cell death event that takes place between 48 and 72 h. Although accelerated somewhat compared to the breast tumor cells, a similar outcome is observed with live cell imaging of the T98G glioblastoma cells (Fig. 7c).

### In situ visualization of the death of putative tumor stem cells as a result of sustained IRES inhibition

Because cells treated with optimal concentrations of cpd_P display no capacity to recover or repopulate, this suggested that putative tumor stem cells, which typically represent the most resilient subset of malignant cells, may also have been eliminated in the course of the population-wide cell death event. To address this issue, a series of experiments were performed in which putative tumor stem cells were identified in situ on the basis of retention of the tracking dye PKH26 [41]. Stock SUM159 cells were stained briefly with the dye and then allowed 1 week (one full passage) to proliferate, during which those cells that divide would distribute the dye equally to their daughter progeny, while putative tumor stem cells, which characteristically divide very infrequently, would remain brightly stained with the non-toxic membrane dye. Pece et al., [41] have shown that PKH26 dye retention can be used to visualize and distinguish tumor stem cells in situ among the population of more actively proliferating cells. Note that although SUM159 is triple negative, highly undifferentiated, and stem-like in nature, it is still hierarchically organized, and only a small proportion of the cells within the population are actually stem cells [42, 43].

**Fig. 8:**
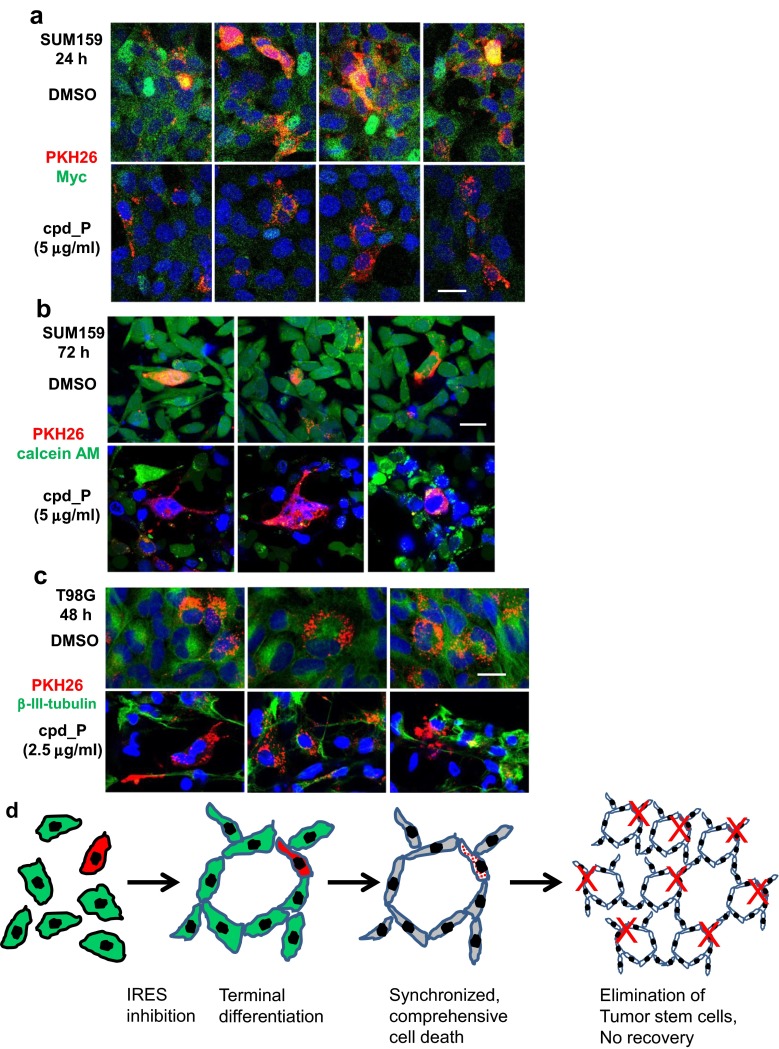
Death of putative tumor stem cells visualized in situ in association with IRES inhibition. **a** SUM159 cells were stained with PKH26 (*red*), washed extensively, and allowed 7 days to propagate and distribute (dilute) the dye to daughter cells at each mitotic event. Cells were then seeded onto 8-well chamber slides, allowed 48 h to recover and resume proliferation, then treated with cpd_P (or vehicle control) in full serum at 5 μg/ml for 24 h. Cells were fixed, permeabilized (using saponin in place of Triton X-100 to protect PKH26), and stained for Myc (*green*) and DAPI. Fields containing *bright red*-stained candidate tumor stem cells were imaged, and four such representative fields are shown for each condition. Overlap of green and red fluorescence produces a yellow signal (i.e., a stem cell with high Myc content). **b** PKH26 (*red*)-stained SUM159 cells described earlier were seeded in 8-well chambered coverslips, allowed 48 h to recover and resume proliferation, then treated in full serum-containing media with cpd_P at 5 μg/ml or DMSO control for 72 h. Calcein AM and Hoescht 33258 were added to the media, and live cell images captured, concentrating on fields containing bright red-stained stem cell candidates. Three such representative fields are shown for each condition. **c** T98G cells were stained with PKH26 (*red*), and allowed 7 days to propagate and distribute the dye to daughter cells, then seeded onto 8-well chamber slides, allowed 48 h to recover and resume proliferation, and treated with cpd_P (or vehicle control) at 2.5 μg/ml for 48 h in low (0.5 %) serum media. Cells were stained for β-III tubulin (*green*) and Hoescht 33258. Fields containing bright red-stained candidate tumor stem cells were imaged, and three such representative fields are shown for each condition. All settings, including PMT voltage, laser power, and pinhole diameter were held constant during image capture of related samples, except for calcein AM, which required constant adjustment in accordance with real-time variations in metabolism by live cells. All *scale bars* 25 μm. **d** A working model for the phenotypic consequences of sustained IRES inhibition in malignant cells. Initially, the tumor cells exhibit no structural organization or polarization and are haphazardly arranged on the growth surface. Continuous exposure to the IRES inhibitor induces terminal differentiation (or a phenotypic alteration closely resembling terminal differentiation), as a result of perpetual inability to synthesize certain key gene products (e.g., Myc) necessary to maintain the undifferentiated phenotype. A marked gain in structural organization is readily appreciated. Cell death is triggered suddenly *i* upon restoration of normal homeostatic mechanisms which detect the underlying abnormalities that cause the cells to be malignant, or *ii* perhaps because these otherwise highly undifferentiated malignant cells may not tolerate the transition to a fully differentiated state, or *iii* alternatively because such an outcome is an integral part of execution of a program related to cornification, where terminal differentiation and cell death are inherently linked. Once triggered, cell death is rapid and comprehensive, resulting in massive loss of viability affecting the entire tumor cell population. Cell death is atypical, with the network of dead cell corpses remaining structurally intact. Tumor stem cells (denoted by *red color*) are eliminated along with the bulk of the tumor cell population, consequently, there is no capacity to rebound and repopulate

At 24 h, the vehicle-treated control fields exhibit multiple bright red candidate stem cells, some of which are intensely stained for Myc themselves (i.e., yellow), or are otherwise surrounded by intensely green-(Myc)-stained cells in close proximity (Fig. 8a). However, for the cells treated with cpd_P, although viability is not yet compromised, a dramatic loss of Myc protein from both the candidate stem cells themselves, as well as the cells surrounding them is observed. As Myc is closely tied to stem cell status [44–46], it is conceivable that this depletion of Myc from the stem cells and their immediate neighbors may undermine the undifferentiated nature and/or compromise the survival of the stem cells. At the 72-h time point, although viable candidate tumor stem cells can still be seen in the control fields, essentially all such bright red stem cell candidates treated with cpd_P are dead, displaying marked chromatin condensation and no esterase-positive cytoplasm (Fig. 8b).

Death of candidate tumor stem cells is also observed in situ when T98G cells are treated with the IRES inhibitor (Fig. 8c). Although it appears that the tumor stem cells are dying in roughly the same time frame and same manner as the bulk of the tumor cell population, a more complete understanding of these events and relationships, i.e., whether it is the tumor stem cells that drive the differentiation program and trigger the population-wide cell death event, or whether the bulk tumor cells are the primary targets of IRES inhibition, with the tumor stem cells merely becoming integrated into the cellular network and eliminated alongside their progeny, will require further investigation.

## Discussion

### Atypical mode of cell death induced by sustained IRES inhibition

A working model for the phenotypic consequences of sustained IRES inhibition (terminal differentiation **→** synchronized cell death) is illustrated in Fig. 8d. Sustained IRES inhibition culminates in massive loss of viability of the malignant cells, yet the response to cpd_P is not like that of a typical cytotoxic agent. Cell death is preceded by dramatic alterations in cytoskeletal organization and tissue architecture, including marked gains in polarity and intercellular connectivity, and formation of tight junctions or neuronal processes, all of which are characteristics of non-malignant cells. Furthermore, the changes induced by cpd_P are completely reversible until a critical threshold in duration of exposure is reached. The formation of extensive physical interconnections between the cells is a key feature and likely very important for mediating both the dramatic phenotypic alteration as well as the population-wide cell death event that follows. The fact that loss of viability is so sudden and affects essentially the entire tumor cell population all within the same time frame distinguishes this mode of cell death from typical stochastic processes such as apoptosis, necrosis, or autophagy, where cell death decisions are made at the level of the individual cell. This phenotypic outcome is even more atypical in that it takes place concomitantly with a marked decrease (rather than gain) in p21 and p27. The Hill slope of the dose-response curve is a reflection of drug mechanism of action, and a Hill slope > ±1 is usually accounted for (in classical enzymology and pharmacology) by cooperativity [23]. It appears in the case of IRES inhibition (with Hill slopes in the −15 to −35 range) that cooperativity is taking place *at the cellular level*. Cell death is non-apoptotic, as PARP cleavage is not observed, caspase 3/7 activation is limited, and no protection afforded by addition of caspase inhibitor Z-VAD-fmk prior to loss of viability. Even upon cell death, catabolism of cellular structural elements is limited, loss of cell membrane integrity is delayed, and cellular disintegration does not occur.

Thus, the atypical mode of cell death induced by sustained IRES inhibition does not fit any of the classical programmed cell death outcomes. Instead, this unique phenotypic outcome most closely resembles cornification, a well-established physiological program in which terminal differentiation is inherently linked with loss of viability [47]. Mailleux et al. have shown that mammary epithelial cells are capable of utilizing squamous differentiation or cornification as an alternative mode of cell death when apoptosis is blocked [48], demonstrating that this mechanism is not restricted to keratinocytes. Cornification involves a reduction of cellular water content, limited proteolytic remodeling of cellular components, and leaves a structurally intact network of non-viable cells, all features in common with the breast tumor and glioblastoma cells subjected to IRES inhibition. In fact, during execution of the cornification program, premature death of the cells prior to completion of differentiation is suppressed by anti-apoptotic and anti-necrotic mechanisms [49]. This could explain the de-induction of BiP and CHOP, and the apparent failure to respond to stress observed in the cells treated with optimal differentiation-inducing concentrations of the IRES inhibitor.

### Potential therapeutic ramifications of IRES inhibition

Together, these findings support the concept that IRES-mediated translation is critical for maintenance of the undifferentiated phenotype, and that selective interference with IRES function has the potential to induce terminal differentiation and trigger population-wide death in otherwise highly undifferentiated malignant cells. Induction of terminal differentiation as a means to eradicate malignant cells is an accepted paradigm for treatment of certain types of leukemia [50, 51]. The results presented here suggest that such a strategy might also be effective in at least some solid tumors as well, and that IRES inhibition may be one way to accomplish this.

The hierarchical organization of tumor cells implies that bidirectional communication or quorum-sensing type mechanisms must be utilized to regulate the equilibrium between tumor stem cells and their progeny comprising the bulk tumor cell population. IRES inhibition apparently perturbs this equilibrium, resulting in synchronous alterations to phenotype affecting the entire tumor cell population. In fact, our results are in line with projections generated using mathematical models simulating the consequences of modulating the frequency with which tumor stem cells undergo symmetrical versus asymmetrical cell division [27].

The molecular and phenotypic consequences of IRES inhibition are complex. Successful implementation of IRES inhibition as a therapeutic strategy will need to take into account the unique pharmacodynamic properties of these compounds. For instance, because the IRES inhibitors are not typical cytotoxic agents, but operate instead by modulating gene expression at the translational level, the highest dose may not be the most effective. Based on the current data, it would appear that this particular strategy of using IRES inhibition to induce terminal differentiation might work best as an initial treatment regimen, intended to eliminate tumor stem cells and the bulk tumor population in parallel, rather than following surgery or cytotoxic chemotherapy, when the goal is to eradicate small numbers of disseminated malignant cells.

One of the long-range goals for development of these prototype IRES inhibitors as useful drugs would be widening of the concentration range at which optimal terminal differentiation and tumor cell death are obtained, through chemical optimization of the lead compound. Yet, the inordinately steep dose-response curves associated with the IRES inhibitors, in which IC_50_ and IC_99_ are achieved at nearly the same concentration, may provide unique pharmacodynamic benefits over agents with more typical dose-response relationships, where achieving IC_99_ may be difficult.

Heterogeneity of the tumor cell population is a well-recognized problem [52], and thought to be a major contributor to therapeutic failure and disease recurrence [53, 54]. It is difficult to achieve complete eradication due to cell-to-cell variability in response. Xia et al. [55] have argued that in terms of therapeutic agents capable of killing tumor cells, the focus should be not on what happens to the “average” cell or the dominant subset, but on the variance among the population. The apparent homogeneity in response to optimal differentiation-inducing concentrations of cpd_P suggests that there may be a tangible kinetic advantage to synchronized over stochastic tumor cell death, and that this might be one way to combat inherent intratumoral heterogeneity.

In total, we have tested 17 different human tumor cell lines representing multiple tumor types, and found the synchronized comprehensive mode of cell death described here to be induced in 5 of them (∼30 %), including (in addition to SUM159 and T98G) an osteosarcoma (143B, Suppl. Fig. 2e, f), as well as another triple-negative breast carcinoma and an acute myeloid leukemia (BT-20, THP-1, data not shown). Some of the other cell lines tested exhibited more acute cell death responses to IRES inhibition (MDA-MB-231 triple-negative breast carcinoma, NIH:OVCAR-3 ovarian carcinoma, SUM149 inflammatory breast carcinoma, Saos-2 osteosarcoma), while a minority of tumor cell lines were highly resistant to cpd_P (Rh30 rhabdomyosarcoma).

The pattern of response noted in ER-positive breast tumor cell lines (e.g., T47D, ZR-75-1) was particularly informative. We find that exposure to cpd_P forces the ER-positive breast tumor cells to shift to a more differentiated phenotype, but this is not associated with extensive loss of viability. In fact, the viability data for the ER-positive cell lines are nearly superimposable with that of the normal human mammary epithelial cells (presented in Suppl. Fig. 2c, d). Importantly, however, we also find that clonogenic survival and mammosphere formation by the ER-positive cell lines are potently inhibited by cpd_P. These assays measure the impact of IRES inhibition on the small fraction of tumor cells with colony-forming or mammosphere-forming capacity, i.e., putative tumor stem cells. Thus, while the bulk of the tumor cell population (already moderately differentiated) is relatively resistant, the tumor stem cells underlying ER-positive breast cancer, which are actually ER-negative [56] and highly undifferentiated, appear to be highly sensitive to IRES inhibition (manuscript in preparation). Of note, death of the tumor stem cells associated with ER-positive breast cancer cell lines in response to IRES inhibition does not appear to be synchronized, nor exhibit features of terminal differentiation, and is favored by low rather than high cell density.

Thus, the synchronized comprehensive mode of cell death is not an invariant outcome of IRES inhibition in all human tumor types. Rather, the data indicate that IRES inhibition has the capacity to induce terminal differentiation and population-wide cell death in susceptible cells under the appropriate conditions. Ultimately, it will be important to characterize the factors which cause certain tumors to be susceptible to triggering of synchronized comprehensive cell death in response to IRES inhibition, and to establish specific biomarkers which can be used to distinguish those tumors.

## Conclusion

The identification of the prototype IRES inhibitors allows us to begin to dissect the relationship between IRES-mediated translation and the malignant phenotype, and begin to determine what might be accomplished by pharmacologically modulating this specialized mode of protein synthesis. We have learned that in susceptible cells at optimal intermediate concentrations of cpd_P, a population-wide phenotypic alteration resembling terminal differentiation is induced, followed closely by a synchronized comprehensive cell death event which eliminates all or very nearly all of the tumor cells. At higher concentrations of the IRES inhibitor, factors such as IGF1R and connexin 43 may be lost, BiP and CHOP are induced, terminal differentiation is incomplete, and the synchronized comprehensive cell death event is not triggered, but is replaced instead by a more progressive, stochastic loss of viability affecting individual cells. Further investigation will be needed to identify precisely all of the factors involved in mediating this outcome, but as a first approximation, it appears the synchronized comprehensive mode of cell death is the result of an intrinsically synthetic lethal combination of changes in IRES-driven proteins (including but not limited to Myc) whose translation is altered by the small-molecule inhibitor.

## Electronic supplementary material

ESM 1 (PDF 2074 kb)
