# Supplementary material for: IRES inhibition induces terminal differentiation and synchronized death in triple-negative breast cancer and glioblastoma cells
Source: Tumour Biol. 2016 Jul 26;37(10):13247–64. doi: 10.1007/s13277-016-5161-4 (PMC5097113; doi:10.1007/s13277-016-5161-4)
Supplement: Supplementary file 1 — (PDF 2074 kb) [file 13277_2016_5161_MOESM1_ESM.pdf]

IRES inhibition induces terminal differentiation and synchronized death in triple-negative breast cancer and glioblastoma cells

Tumor Biology

Christos Vaklavas, William E. Grizzle, Hyoungsoo Choi, Zheng Meng, Kurt R. Zinn,
Kedar Shrestha, and Scott W. Blume

University of Alabama at Birmingham

scott.blume@ccc.uab.edu

Supplemental Figure Captions:

Supplemental Fig. 1 Sustained IRES inhibition results in massive loss of viability in triple-negative breast carcinoma cells.

(a) Viability of SUM159 cells treated with cpd_P assessed using ATP (CellTiter-Glo) as endpoint (as in Figure 1A), plotted on a standard scale. **(b)** Viability of SUM159 cells treated with cpd_P assessed using cytoplasmic protease (Promega) as endpoint. Separate readings are obtained for protease activity recovered from intact adherent cells (solid lines) versus that measured in media overlying cell monolayer (dotted lines, indicative of loss of cell membrane integrity accompanying cell death). **(c)** Concordance of three measures of cell viability: MTT, ATP, and cytoplasmic protease, in terms of cpd_P dose-response (120 h endpoint) in SUM159. **(d)** Graph comparing three measures of viability over time in cells treated with optimally effective concentration of cpd_P (5 μg/ml) in SUM159. **(e)** Assessment of ATP (solid bars) and ADP (striped bars) levels in SUM159 cells exposed to cpd_P for 48, 72, or 96 hours. Results are plotted relative to ATP levels in vehicle-treated cells (= 100%). **(f)** Washout / recovery assay for SUM159 cells (as in Figure 1C) performed under Low serum (0.5% FCS, no supplemental insulin) conditions. **(g)** Viability of SUM159 breast tumor cells was assessed following treatment with increasing concentrations of IRES inhibitor cpd_P or analog P-2 or P-3 for 72 h under Low serum (0.5%) conditions (all data + standard error). **(h)** Viability of T98G glioblastoma cells was assessed following treatment with increasing concentrations of IRES inhibitor cpd_P or analog P-2 or P-3 for 72 h under Low serum (0.5%) conditions (all data + standard error).

Supplemental Fig. 2 Sustained IRES inhibition results in massive loss of viability in glioblastoma cells, yet normal cells tolerate prolonged exposure to the IRES inhibitor.

(a) Viability of T98G cells treated with cpd_P assessed using cytoplasmic protease as endpoint. All data ± SEM.

(b) Results of washout / recovery assay performed on T98G cells under Low serum conditions. Viability is measured using ATP as endpoint, and results plotted on logarithmic scale. The paired bars allow direct comparison of % viable cells remaining at end of treatment period (72/0) versus % viable cells remaining at end of recovery period (72/72). All data $\pm$ SEM. **(c, d)** Normal primary human mammary epithelial cells (HMECs) were treated with IRES inhibitor cpd_P (or vehicle control) at varying concentrations for 72 h, under Full (growth factor supplemented) or Low (growth factor deprived) conditions. Cell survival was assessed using ATP as endpoint and presented relative to the cell number at initiation of treatment (time 0=100%). All data $\pm$ SEM in triplicate. **(e, f)** 143B human osteosarcoma cells were treated with IRES inhibitor cpd_P (or vehicle control) at varying concentrations for 72 h, under Full serum or Low serum conditions. Cell survival was assessed using ATP as endpoint and plotted on a logarithmic scale. All data $\pm$ SEM.

Supplemental Fig. 3 IRES inhibition induces marked gains in structural organization and intercellular networking of breast tumor cells. α-tubulin staining patterns for SUM159 breast tumor cells treated under Low serum conditions (0.5% FCS, no supplemental insulin) for 24 h with cpd_P at the indicated concentrations. Scale bar 50 μm.

Supplemental Fig. 4 Titration of cell density and its effect on susceptibility to IRES inhibition.
(a) SUM159 breast tumor cells were seeded at varying densities, allowed 48 h to recover and resume proliferation, then treated with IRES inhibitor cpd_P at varying concentrations for 72 h (as shown graphically in Figure 4A). Phase contrast images and % viable cells (relative to vehicle-treated control seeded at same density) are shown. Red boxes indicate synchronized, comprehensive cell death attained when cell density exceeds threshold. Green box indicates resistant state of cells seeded below threshold. Scale bar 100 μm. **(b)** SUM159 or T98G cells were seeded at 3% or 2.25% respectively (below threshold), allowed 48 h to recover and resume proliferation, then treated with cpd_P at optimal intermediate concentration (5 μg/ml for SUM159; 7 μg/ml for T98G) for 144 h continuously, or else left untreated for 72 h followed by 72 h treatment. Cells treated at low density remain completely viable, undergoing only modest growth inhibition in response to cpd_P. However, if cells seeded at low density are allowed to reach higher density before treatment is initiated, they become susceptible to comprehensive cell death triggered by IRES inhibition.

Supplemental Fig. 5 ***De novo*** **establishment of ZO-1-positive tight junctions in breast tumor cells subjected to IRES inhibition.** SUM159 breast tumor cells were treated with cpd_P at 2.5 or 5 μg/ml for 48 h under Low serum conditions, then stained for ZO-1. Scale bar 25 μm.

Supplemental Fig. 6 Molecular correlates of tumor cell death induced by IRES inhibition in SUM159 breast tumor cells and T98G glioblastoma cells. **(a)** Composite western blot results obtained from whole cell lysates prepared from SUM159 cells treated with varying concentrations of cpd_P for 24, 48, or 72 h under Low serum (0.5% FCS, no supplemental insulin) conditions. **(b)** Composite western blot results obtained from whole cell lysates prepared from T98G cells treated with varying concentrations of cpd_P for 24, 48, or 72 h under Full serum (10% FCS, 10 μg/ml insulin) conditions.

Supplemental Fig. 7 Induction of CHOP at high but not intermediate concentrations of cpd_P. T98G cells were stained for actin (AlexaFluor 488-conjugated phalloidin, green) and CHOP (red) following 24 h exposure to intermediate or high concentrations of cpd_P (or vehicle control) under Full serum **(a)** or Low serum **(b)** conditions as indicated. The results, which match the western blot data, provide a visual demonstration of how cells treated at the optimal intermediate concentrations of the IRES inhibitor appear not to experience or at least not to perceive translational stress, or have blocked the translational stress response in favor of pursuing the phenotypic shift toward terminal differentiation. Scale bar 25 μm.

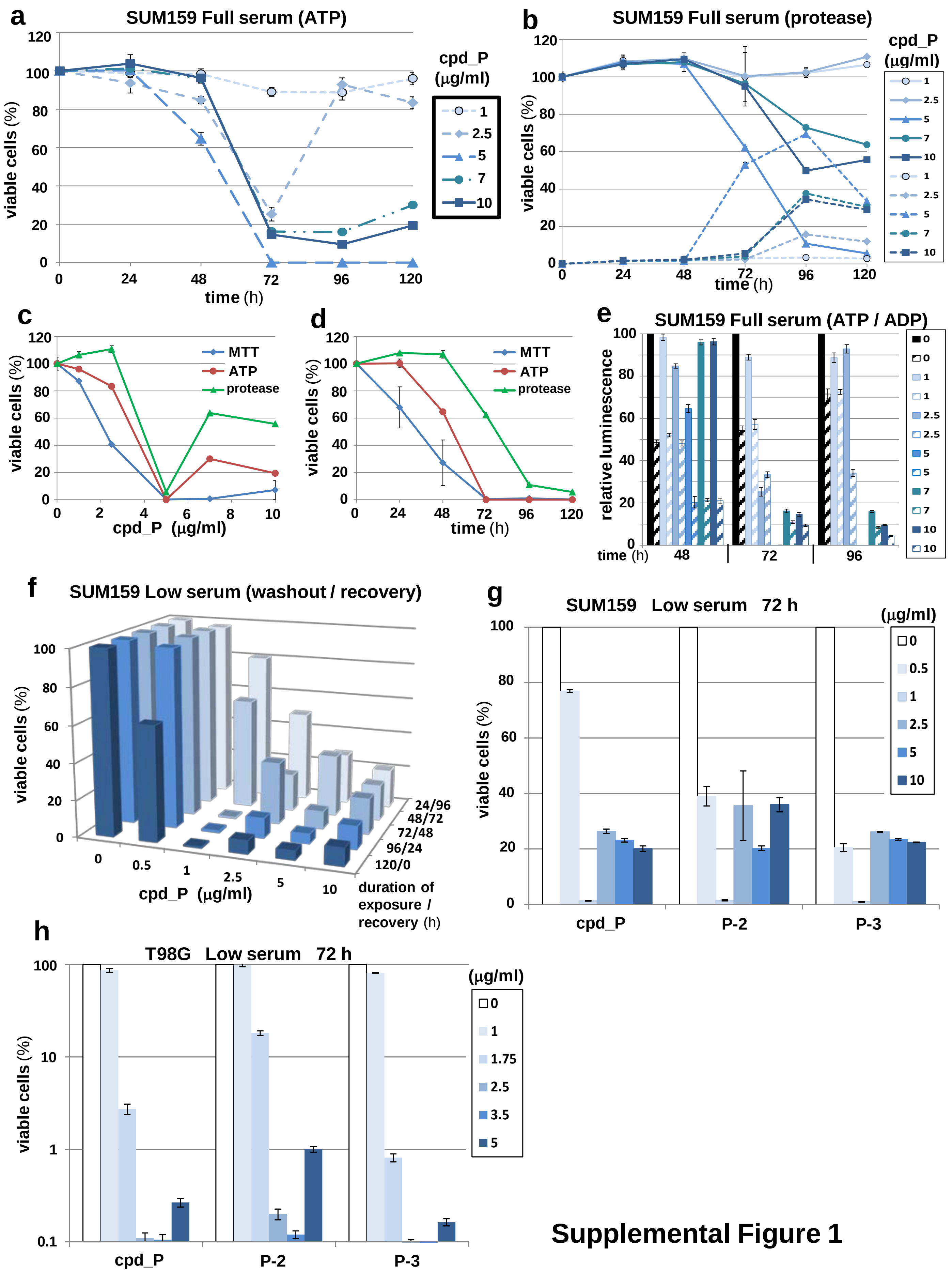

Supplemental Figure 1

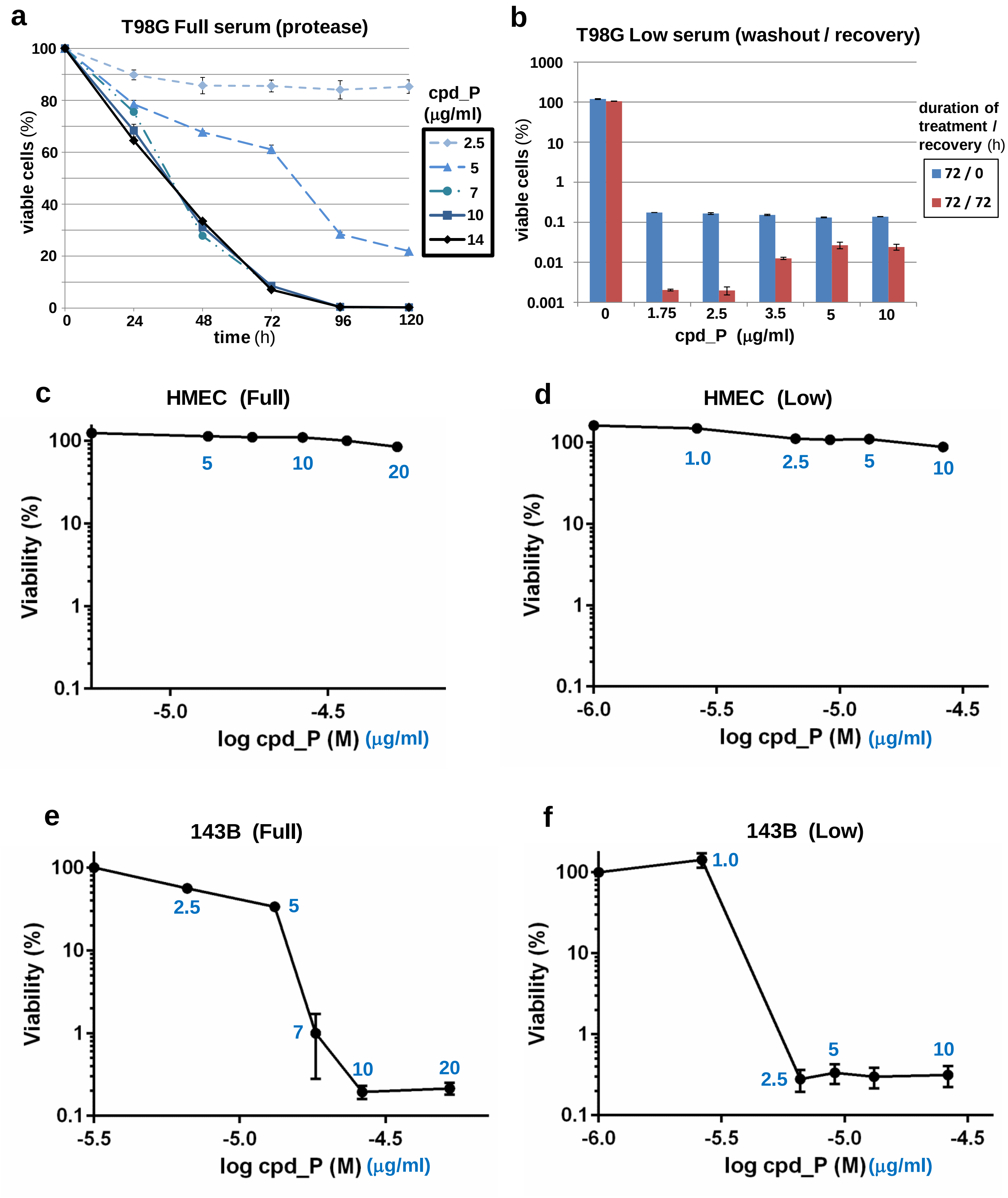

Supplemental Figure 2

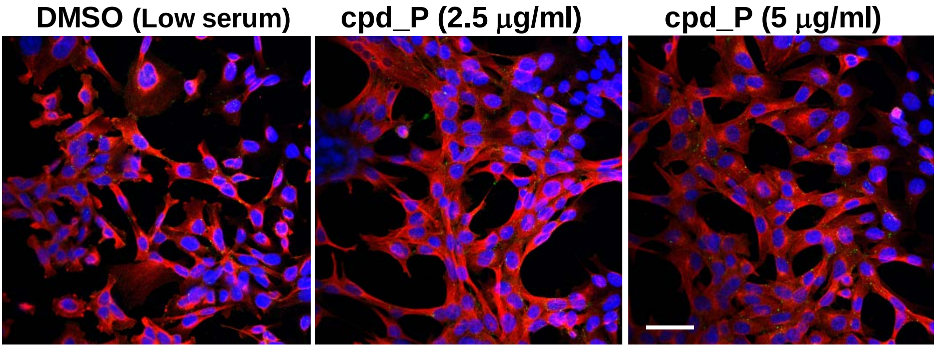

Supplemental Figure 3

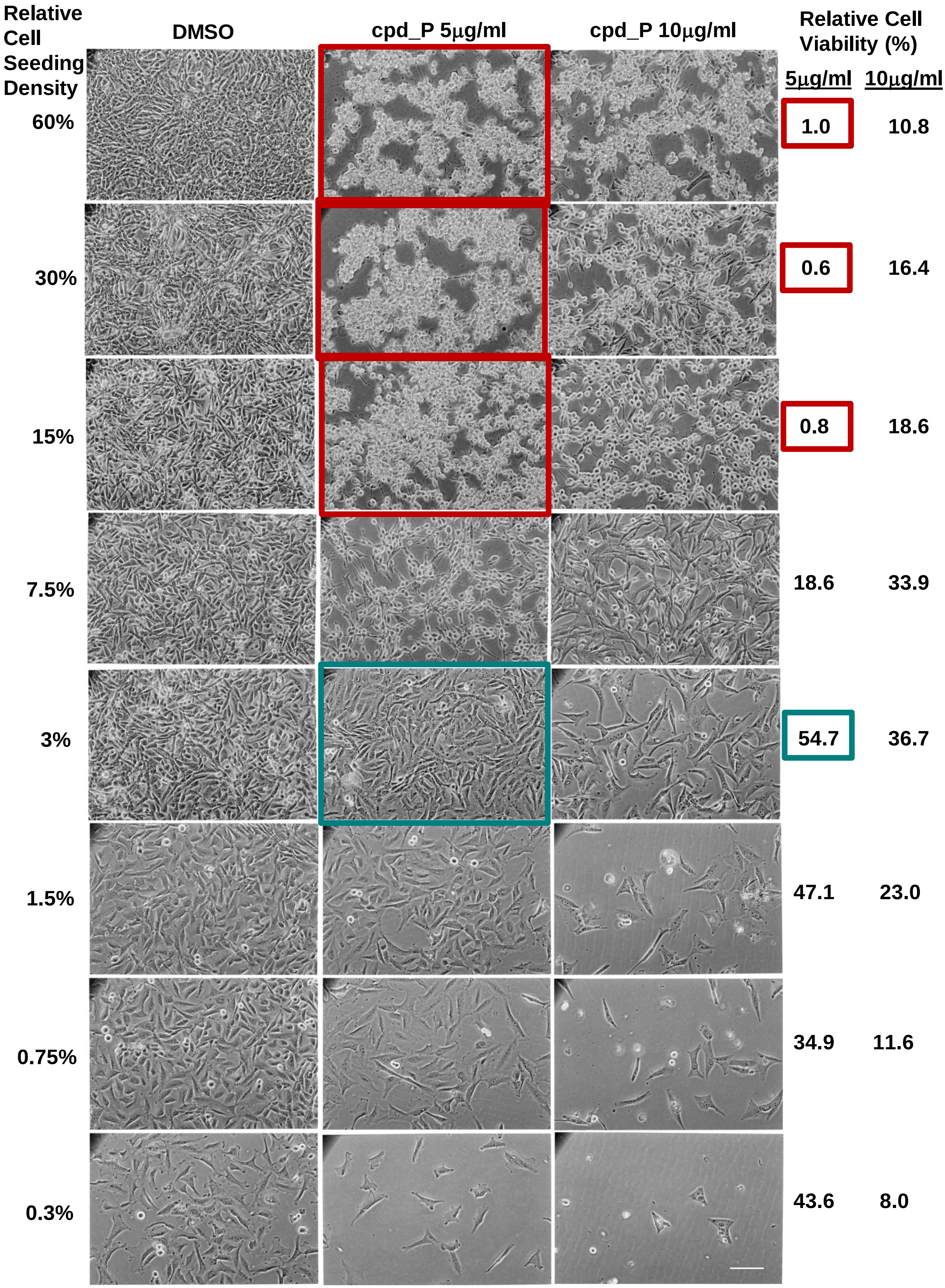

Supplemental Figure 4 a

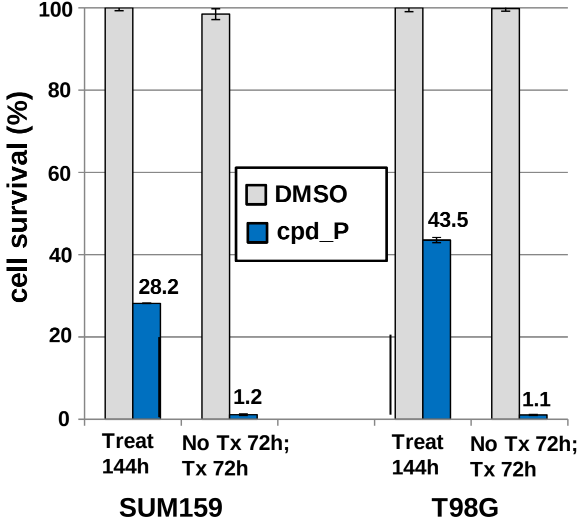

Supplemental Figure 4 b

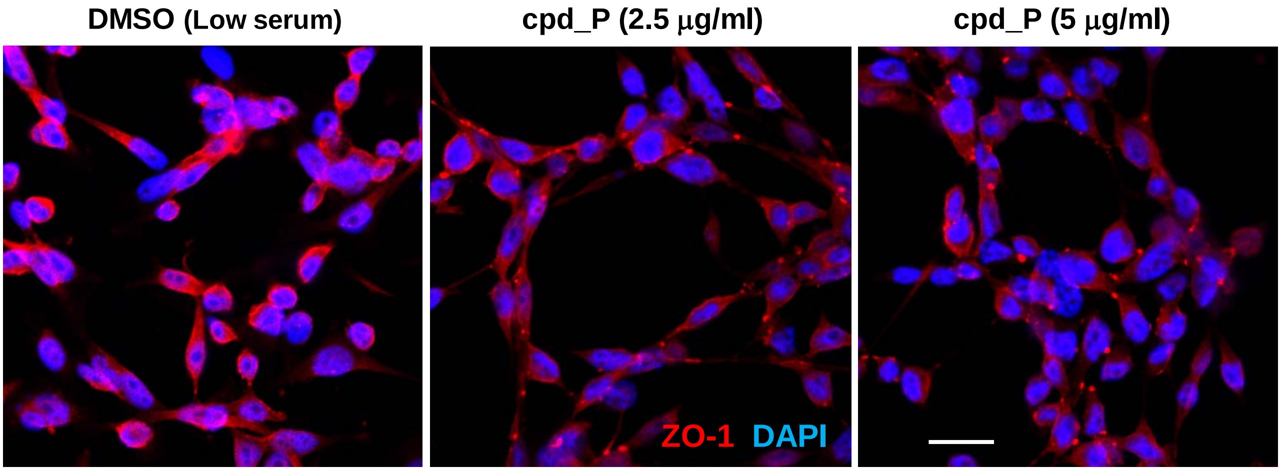

Supplemental Figure 5

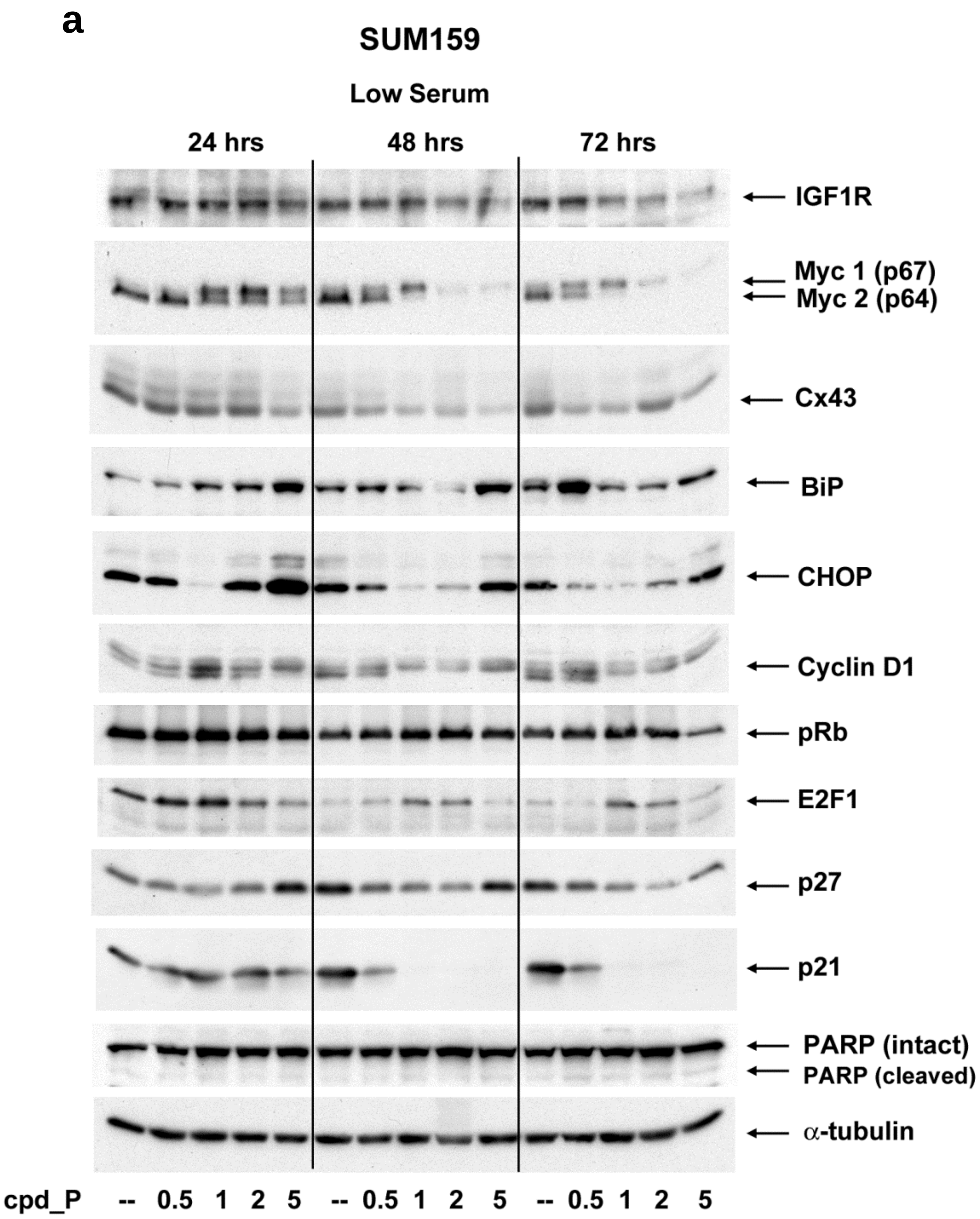

Supplemental Figure 6 a

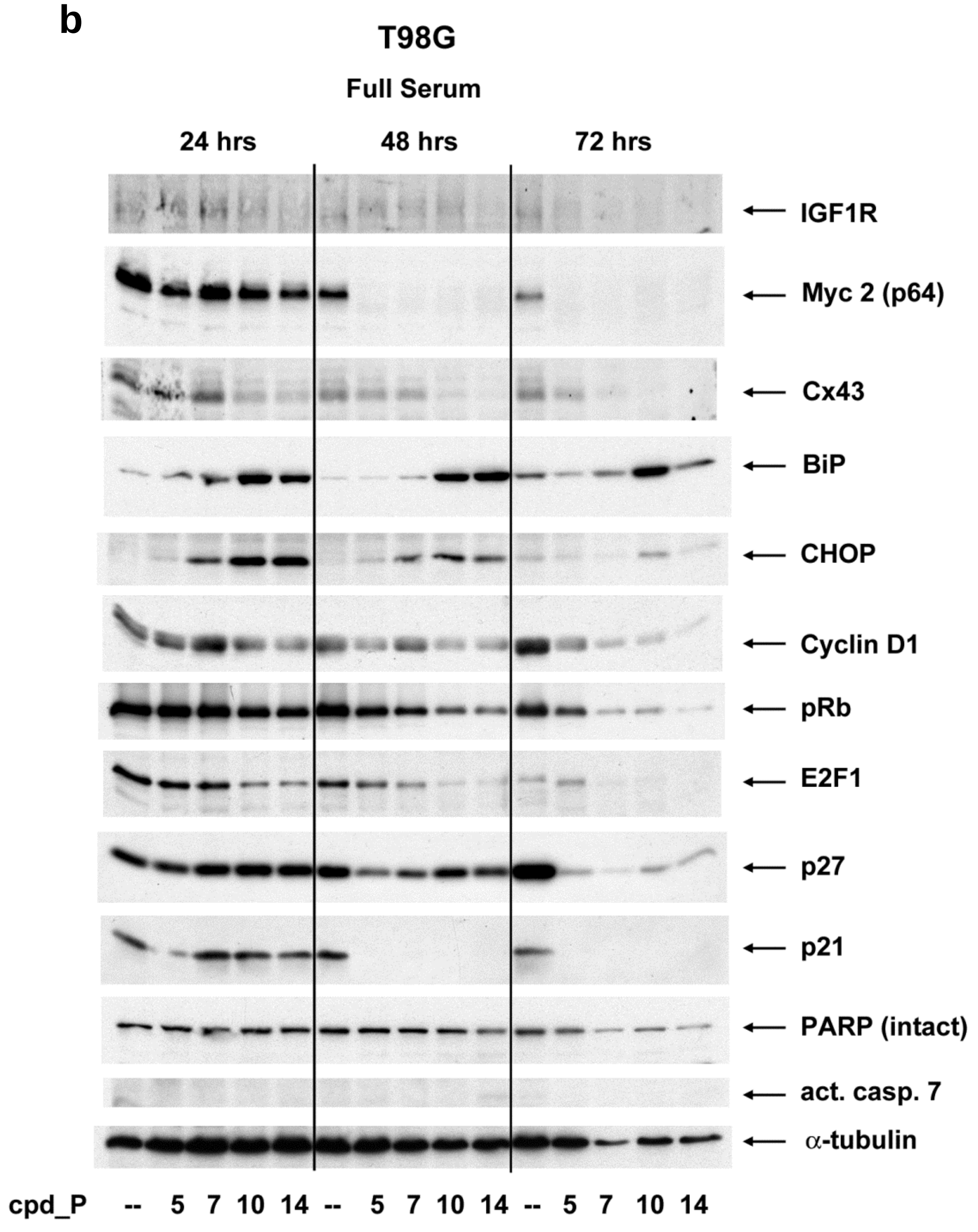

Supplemental Figure 6 b

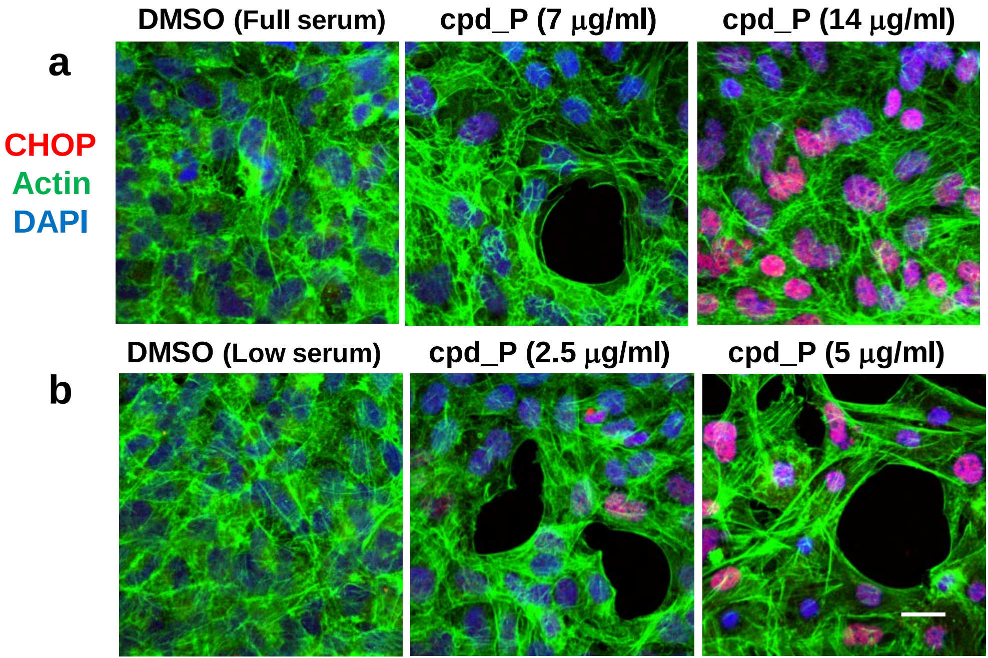

Supplemental Figure 7
